# Transcriptome profiling of *Staphylococci*-infected cow mammary gland parenchyma

**DOI:** 10.1186/s12917-017-1088-2

**Published:** 2017-06-06

**Authors:** Ewa M Kosciuczuk, Paweł Lisowski, Justyna Jarczak, Alicja Majewska, Magdalena Rzewuska, Lech Zwierzchowski, Emilia Bagnicka

**Affiliations:** 1 Department of Animal Improvement, Institute of Genetics and Animal Breeding Polish Academy of Sciences, 36a Postepu str., Jastrzebiec, 05-552 Poland; 2 Department of Physiological Sciences, Faculty of Veterinary Medicine, Warsaw University of Life Sciences, 02-776 Warsaw, Poland; 3 Department of Pre-Clinical Sciences, Faculty of Veterinary Medicine, Warsaw University of Life Sciences, 02-776 Warsaw, Poland; 4 Present address: Robert H. Lurie Comprehensive Cancer Center, Northwestern University, Chicago, IL USA

**Keywords:** Dairy cows, Udder parenchyma, Chronic mastitis, Transcriptomics, Gene expression profiling, microarray, qPCR

## Abstract

**Background:**

Genome-wide gene expression profiling allows for identification of genes involved in the defense response of the host against pathogens. As presented here, transcriptomic analysis and bioinformatics tools were applied in order to identify genes expressed in the mammary gland parenchyma of cows naturally infected with coagulase-positive and coagulase-negative *Staphylococci*.

**Results:**

In cows infected with coagulase-positive *Staphylococci*, being in 1st or 2nd lactation, 1700 differentially expressed genes (DEGs) were identified. However, examination of the 3rd or 4th lactations revealed 2200 DEGs. Gene ontology functional classification showed the molecular functions of the DEGs overrepresented the activity of cytokines, chemokines, and their receptors. In cows infected with coagulase-negative *Staphylococci,* in the 1st or 2nd lactations 418 DEGs, while in the 3rd or 4th lactations, 1200 DEGs were identified that involved in molecular functions such as protein, calcium ion and lipid binding, chemokine activity, and protein homodimerization. Gene network analysis showed DEGs associated with inflammation, cell migration, and immune response to infection, development of cells and tissues, and humoral responses to infections caused by both types of *Staphylococci*.

**Conclusion:**

A coagulase-positive *Staphylococci* infection caused a markedly stronger host response than that of coagulase-negative, resulting in vastly increased DEGs. A significant increase in the expression of the *FOS*, *TNF*, and genes encoding the major histocompatibility complex proteins (MHC) was observed. It suggests these genes play a key role in the synchronization of the immune response of the cow’s parenchyma against mastitis-causing bacteria. Moreover, the following genes that belong to several physiological pathways (KEGG pathways) were selected for further studies as candidate genes of mammary gland immune response for use in Marker Assisted Selection (MAS): chemokine signaling pathway (*CCL2*, *CXCL5*, *HCK*, *CCR1*), cell adhesion molecules (CAMs) pathway (*BOLA-DQA2*, *BOLA-DQA1*, *F11R*, *ITGAL*, *CD86*), antigen processing and presentation pathway (*CD8A*, *PDIA3*, *LGMN*, *IFI30*, *HSPA1A*), and NOD-like receptor signaling pathway (*TNF*, *IL8*, *IL18*, *NFKBIA*).

**Electronic supplementary material:**

The online version of this article (doi:10.1186/s12917-017-1088-2) contains supplementary material, which is available to authorized users.

## Background

In cows, disorders of mammary gland function are most often caused by inflammation from a bacterial infection as the main mastitis pathogens [1]. However, inflammation depends on both environmental factors as well as immune system efficiency [2, 3]. Mechanisms of the immune response depend on different populations of cells and their secreted mediators [4–7]. Expression profile analysis of immune system genes in the mammary gland tissues of both healthy animals and those infected by different pathogens may help explain the specific immune response mechanisms. Furthermore, these analyses could indicate the tissue-specific physiological processes and biochemical pathways involved in different types of infections. Moreover, this knowledge could be essential for the therapy and eradication of mastitis [5]. Microarray technology enables the user to measure vast amounts of gene expression data under different physiological circumstances simultaneously, and, therefore, it allows for the identification of the genes involved in the defense of the host against microbial infection [8]. It also aids in understanding the processes that occur during mastitis [9].

Studies on gene expression profiles in mammary gland tissues and bovine milk leukocytes have been previously performed. Swanson et al. (2009) [10] reported differences in the expression level of several hundred genes in response to an infection by *Streptococcus uberis*, including a large number of genes associated with the immune system. Mitterhuemer et al. [11] studied the influence of *Escherichia coli* on the local (infected quarters) and systemic (neighboring quarters) transcriptome in the mammary glands of dairy cattle. They revealed that local inflammation influenced the expression of genes engaged in the immune response and inflammation, while the systemic response covered the expression of genes involved in antigen processing and presentation, cytokines, protein degradation, and apoptosis. Lützow et al. (2008) [12] identified two main groups of genes (gene clusters) differentially expressed in the mammary gland of cows in response to infection by *Staphylococcus aureus*. The up-regulated genes encoded proteins involved in intracellular signaling, primarily cytokines and chemokines. The genes with lower expression were encoded for extracellular matrix proteins, cellular cytoskeleton proteins, receptors, and intracellular signaling proteins. Moreover, Gilbert et al. [13] found that crude *E. coli* lipopolysaccharide (LPS), the endotoxin of the Gram-negative bacteria, stimulated expression of many more genes than the *S. aureus* infection did. They also stated that LPS induced different mechanisms of leukocyte enrollment.

However, most of these studies were carried out either in vitro on cultured cells derived from mammary gland tissues [10, 13, 14], on experimentally infected animals [10–12], or on milk somatic cells derived from mastitic cows [15]. Moreover, it should be noted that Krappmann et al. [16] proved that mammary gland epithelial cells, obtained from milk somatic cells using magnetic beads, probably did not reflect the metabolic processes proceeding in secretory cells of the mammary gland itself; therefore, they could not substitute the study on the mammary gland biopsies. Other researchers comparing results obtained in vitro and in vivo in different tissues draw similar conclusion in that the in vitro study does not fully reflect the conditions occurring in the living organism [10, 17–19]. Thus, the present study was performed in vivo in animals with recurrent and incurable mammary gland inflammation. The aim of the study was transcriptional profiling and bioinformatics analysis of genes expressed in secretory tissue (parenchyma) derived from dairy cow mammary glands naturally infected with coagulase-positive and coagulase-negative *Staphylococci* in comparison with those of healthy mammary glands.

## Results

The microbiological status of the collected mammary gland samples, as well as information on the average lactose content and milk somatic cell count, was presented in detail previously [20]. Briefly, the microbial status showed that 30% of studied samples were free from bacterial pathogens. In the remaining samples, only bacteria from *Staphylococcus* genus were found. The lowest level of somatic cells was found in milk of healthy cows being in 1st or 2nd lactations and the highest in the milk of cows in 3rd or 4th lactations infected with coagulase-positive *Staphylococci*.

### Comparison of gene expression profiles in coagulase-positive *Staphylococci*-infected and healthy mammary gland parenchyma

Comparing transcript levels in the secretory epithelial tissue of cows infected with coagulase-positive *Staphylococci* in the 1st or 2nd lactation (group CoPS-1/2) and in healthy cows (H) identified 1700 differentially expressed genes (DEGs), meeting the criteria of log FC > 0.5 and *P* < 0.05. Among these genes, 1360 were up-regulated and 340 were down-regulated in bacteria-infected cows. For these genes, 223 biological processes, 85 molecular functions, and 36 cellular component categories were ascribed (Additional files 1 and 2). In the bacteria-infected udders, DEGs were associated with the immune defense response, immune system processes, response to wounding, amino acid transport, transportation of metabolic oxoacids, and ketone metabolic processes. When genes were classified according to their molecular functions, the DEGs were responsible for the activities of chemokines, cytokines and their receptors, protein binding, transmembrane transporter activity, and nucleotide binding. The cellular component genes included those encoding proteins of major histocompatibility complex (MHC) classes I and II. Ten categories of the most differentially expressed genes representing biological processes, molecular functions, and cellular components are shown in Additional file 3: Figure S1. The charts show the categories hierarchized according to increasing *P* values.

A comparison of the transcriptomes of cows from the CoPS-1/2 and H groups identified the network of genes responsible for the migration of immune cells. In this network, the “nodes” represent key genes up-regulated in the CoPS-1/2 group: *MMP7* (matrix metallopeptidase7*)*, *PLAU* (plasminogen activator, urokinase), *ANXA2* (annexin A2), *COL1A1* (collagen, type I, alpha 1), and *TIMP1* (TIMP metallopeptidase inhibitor 1). In Additional file 3: Figure S2, the “cellular movement, hematological system development and function, immune cell trafficking” network is presented with differentially expressed genes and key genes included in this network.

In cows undergoing the 3rd or 4th lactations (CoPS-3/4), 2200 DEGs were identified, meeting the criteria of logFC > 0.5 and *P* < 0.05. There were 1389 up-regulated and 811 down-regulated genes in CoPS-3/4 as compared to H (Additional files 4 and 5). The DEGs were involved in 285 biological processes, 71 molecular functions, and 49 cellular component categories. The biological processes influenced by DEGs included inflammatory responses stimulus responses, lipid biosynthesis, and neutral lipid metabolic processes. The identified molecular functions primarily represented cytokine and chemokine activity as well as their receptors, peptidase activity, magnesium ion binding and oxidoreductase activity. In the cellular component category, genes encoding MHC (major histocompatibility complex) protein systems were identified. Ten categories of biological processes, molecular functions, and cellular components of the most differentially expressed genes are shown in Additional file 3: Figure S3. Comparison of mammary parenchyma transcriptomes of cows from the CoPS-3/4 and H groups revealed four networks of genes which differ in expression level: (A) network of cell metabolism, (B) network of the organization and function of the cells, (C) network of the inflammatory response, and (D) network of the response to infection and development of cells and tissues. Networks A, B, and C contain, respectively, the node (key) genes up-regulated in the CoPS-3/4 group: *FOS* (*Bos taurus* FBJ murine osteosarcoma viral oncogene homolog), *ILβ* (interleukin-β), and *TNF* (tumour necrosis factor). Additional file 3: Figure S4 shows the identified networks with differentially expressed genes.

### Comparison of gene expression profiles in healthy and coagulase-negative *Staphylococci*-infected mammary gland parenchyma

Comparing transcript levels of the mammary gland parenchyma of the CoNS-1/2 group (cows infected with coagulase-negative *Staphylococci*, being in their 1st or 2nd lactations) with the H group identified 418 DEGs, meeting the criteria of logFC > 0.5 and *P* < 0.05. Of these genes, 117 showed increased (up-regulated) and 301 had decreased (down-regulated) expression in animals infected by bacteria. For these genes, 18 biological processes, five molecular functions, and nine cell component categories were identified (Additional files 6 and 7). Biological processes where DEGs were represented included defense response, stress response, antigen presentation, and inflammatory response. In the case of molecular function categories, odorant binding, receptor binding, and RNA binding were highly associated with coagulase-negative *Staphylococci* infection of the mammary secretory tissues. For cellular component categories, DEGs belonged to a group of genes encoding MHC and extracellular matrix proteins. Ten categories of biological processes, molecular functions, and cellular components of the most differentially expressed genes are shown in Additional file 3: Figure S5 according to increasing *P* value. In the CoNS-1/2 group, a network of genes responsible for intercellular signaling, including *EGR1* (early growth response protein 1) and *TGFB3* (transforming growth factor beta 3) as key genes (“nodes”), that represented intragenic network were identified (Additional file 3: Figure S6).

Gene expression analysis profiles of udder parenchyma of cows in their 3rd or 4th lactations, infected with coagulase-negative *Staphylococci* (CoNS-3/4 group vs. H group), identified 1200 DEGs, meeting the criteria of logFC > 0.5 and *P* < 0.05. Among these, 933 genes were up-regulated and 267 were down-regulated in the group infected with bacteria. These genes were involved in 206 biological processes, 56 molecular functions, and 46 cellular component categories (Additional files 8 and 9). In the case of biological processes, genes with differed expression were those engaged in the defense response, immune response, signal transduction, lipid biosynthesis, and organic acid metabolism. As to the molecular function, these genes were responsible for protein binding, chemokine activity, calcium ion binding, protein homodimerization, and lipid binding. Among the cellular component categories, genes encoding cell surface proteins and MHC I proteins were identified. Ten categories of biological processes, molecular functions, and cellular components of the most differentially expressed genes, categorized by increasing *P* value, are shown in Additional file 3: Figure S7.

Genes involved in the response to CoNS infection form five gene networks: (A) cell morphology, (B) organization of the cells, (C) cell proliferation, (D) reaction to tissue injury, and (E) humoral response (Additional file 3: Figure S8). In these networks, several “node” genes were distinguished: *UBC* (ubiquitin) for network A, *FOS* for network B, *TNF* for network C, *SOD2* (superoxide dismutase 2) for network D, and *IL2R* (interleukin-2 receptor) and *PTGS2* (prostaglandin endoperoxide synthase-2) for network E.

### KEGG biochemical pathway analysis

To determine over-represented biochemical pathways, including genes up-regulated during *Staphylococci* infections, the Kyoto Encyclopedia of Genes and Genomes (KEGG) database was searched (Table 1). In cows from the CoNS-1/2 group several pathways associated with the body’s response to the presence of bacteria were identified. For all pathways, the differences between infected and non-infected udders were significant at *P* < 0.05. Importantly, all identified pathways were associated with immunological processes. A comparison of groups CoPS-1/2 vs. H identified 10 pathways, including genes involved in the immune response. Analysis of CoPS-3/4 vs. H groups identified 12 pathways and CoNS-3/4 vs. H revealed 11 pathways. For all three comparisons, changes in pathways of complement and coagulation cascades, cytokine-cytokine receptor interaction, antigen processing and presentation, leukocyte trans-endothelial migration, nucleotide oligomerization domain (NOD)-like and Toll-like receptors, chemokine signaling and cell adhesion molecules (*CAMs*) were identified.

**Table 1:** Pathways and number of genes up- or down-regulated in CoPS-1/2, CoPS-3/4, and CoNS-3/4 bovine groups

Pathway	No. of genes		
	CoPS-1/2	CoPS-3/4	CoNS-3/4
Complement and coagulation cascades	22	21	18
Cytokine-cytokine receptor interaction	31	19	22
Chemokine signaling pathway	25	25	18
Antigen processing and presentation	13	19	18
NOD-like receptor signaling pathway	11	15	10
Cell adhesion molecules (CAMs)	16	24	14
Leukocyte trans-endothelial migration	16	22	12
Toll-like receptor signaling pathway	13	14	11
Gap junction	12	–	–
T cell receptor signaling pathway	–	17	–
Natural killer cell mediated cytotoxicity	–	15	–
Immune network for IgA production	12	12	10
Cytosolic DNA-sensing pathway	–	8	–

**Fig. 1:**
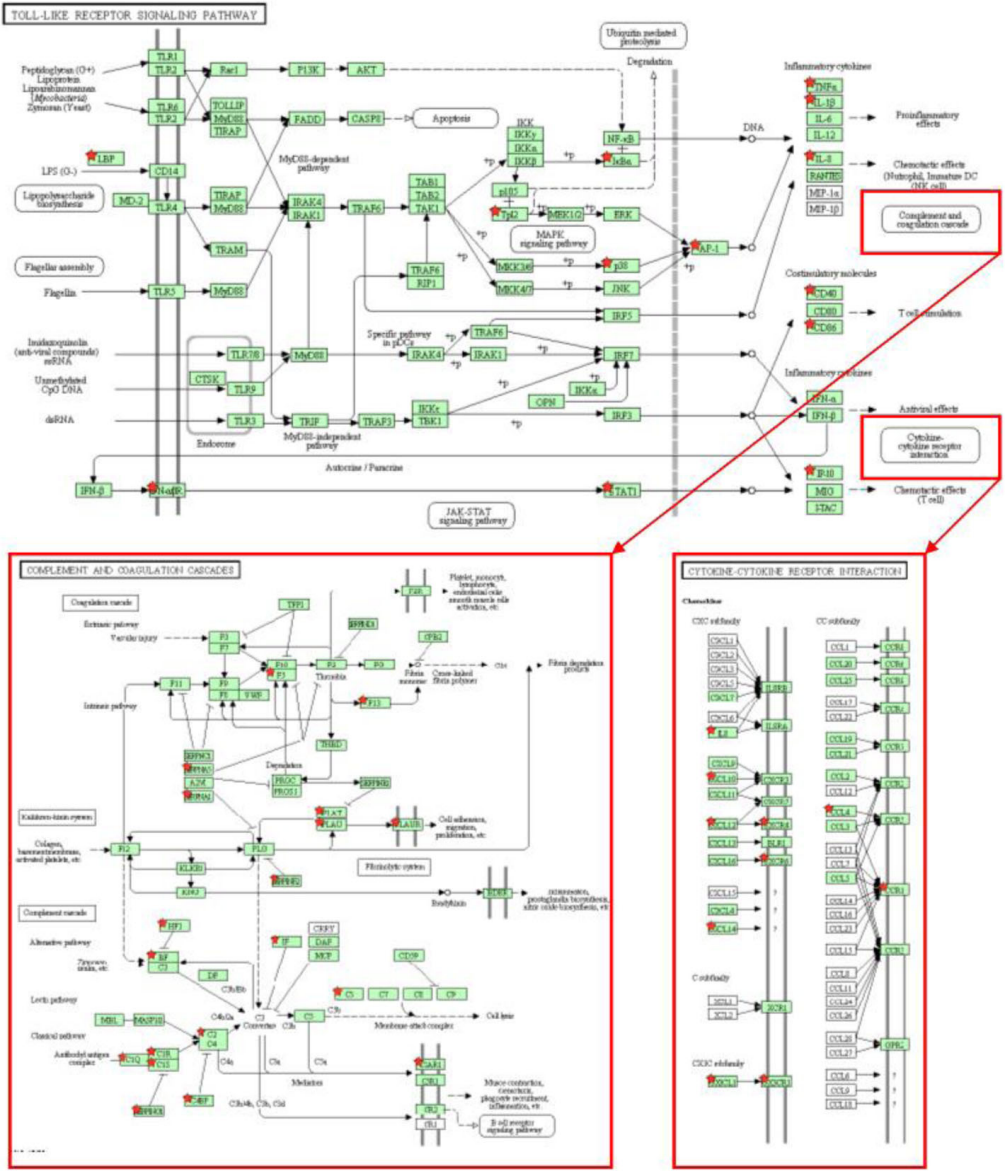
Gene map of the signaling pathways of the Toll-like receptor, activation of complement and interaction of cytokines (shown only partly) derived from the KEGG database. Genes showing significantly different expression in the CoPS-3/4 vs. H groups are marked with red asterisks. The figure shows the dependence of complement activation and cytokine pathways on the Toll-like receptor pathway (*red arrows*). Differentially expressed genes are located on the paths leading to the complement and cytokine pathways

In pathways associated with the response of the mammary gland secretory tissues to the bacteria, approximately 10 to 30 DEGs were found, making the detected paths highly significant (with *P*-values ranging from 5 × 10^−2^ to 5 × 10^−9^). Figure 1 shows an example diagram of the signaling pathway of a Toll-like receptor together with the selected DEGs comparing the CoPS-3/4 vs. H groups. The figure shows the dependence of the complement cascade and cytokine pathways on the Toll-like receptor pathway. DEGs in the CoPS-3/4 vs. H comparison were involved in metabolic reactions leading to complement and cytokine pathways.

In the CoPS-1/2 group, intestinal immune network for IgA (immunoglobulin A) production, gap junction, and hematopoietic cell lineage pathways were involved in the immune response to infection. In the CoPS-3/4 group, genes associated with the lysosome, T cell receptor signaling pathway, natural killer cell mediated cytotoxicity, and cytosolic DNA-sensing pathways were differentially expressed as compared to the H group.

### Identification of functional gene clusters

Bioinformatics analysis of microarray results showed that the identified DEGs were involved in multiple biological processes. Clustering analysis using the DAVID Functional Clustering Module allowed for a more accurate classification and interpretation of results. Several clusters were identified in each of the transcriptomes. Functional clustering showed that identified clusters were primarily associated with immunological processes (Additional file 10). Analysis of samples collected from cows assigned to the groups CoPS-1/2, CoPS-3/4, CoNS-1/2, and CoNS-3/4 showed six, eight, four, and five clusters, respectively. These clusters contained groups of genes associated with the immune response, antigen presentation, MHC proteins, chemotaxis of leukocytes, leukocyte adhesion, activity of chemokines and cytokines, complement activation, humoral response, B-cell activity, and activity of immunoglobulins. Furthermore, in samples taken from the CoPS-3/4 group, a cluster No. 4, consisting of genes associated with metabolism and biosynthesis of lipids and fatty acid was identified. In samples derived from the CoNS-1/2 animals, three additional clusters were identified and were associated with the serine-threonine kinase receptor signaling pathway, transforming growth factor beta (TGF-β), and a cluster of genes related to the organization of cytoskeletal actin.

### Validation of selected genes by real-time quantitative PCR (qPCR)

**Fig. 2:**
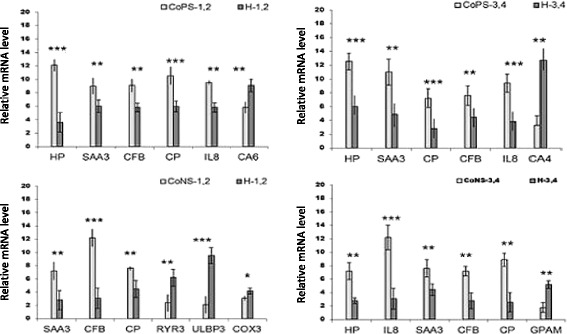
Validation of microarray analysis using real-time PCR. The relative levels of mRNA expressed are shown as the mean (with standard error – SE) of six animals. Groups differ from each other significantly at *P < 0.05, **P < 0.01, and ***P < 0.001

Validation of microarray results was performed to confirm the accuracy of the transcriptomic analysis for each of the four comparisons using individual, unpooled mRNA samples. The relative transcript levels of validated genes were compared between infected and non-infected samples within age groups separately (lactations 1/2 and 3/4). In concordance with the microarray results, the levels of *SAA3* (serum amyloid A3), *CFB* (complement factor B), and *CP* (cytoplasmic polyadenylation) were higher in both CoPS and both CoNS group samples. Moreover, comparisons between CoPS-1/2 and H-1/2 groups and CoPS-3/4 and H-3/4 groups revealed that, in both CoPS groups, *HP* (haptoglobin) and *IL8* (interleukin 8) gene transcripts were higher than in H groups, while the levels of *CA6* (Carbonic anhydrase 6) and *CA4* (Carbonic anhydrase 4) were higher in H-1/2 and H-3/4 groups, respectively. In the CoNS-3/4 group, *HP* gene expression was higher than in H-3/4 group, similar to both CoPS groups. Patterns of gene expression in cow mammary parenchyma, as measured with real-time PCR (qPCR) between particular groups (CoNS and CoPS vs. H) according to lactation status, are shown in Fig. 2.

## Discussion

In the present study, hundreds of genes are differentially expressed in the parenchyma derived from infected cows’ mammary glands as compared to healthy ones. The highest number of DEGs, 2200 genes, was identified in CoPS-3/4 group. The infection caused by coagulase-negative *Staphylococci* (CoNS) influenced a smaller number of genes in this age group – 1200. Cows in lactations 1/2 were also characterized by different gene expression profiles; the numbers of DEGs were 1200 and 418 in the CoPS and CoNS groups, respectively. These results reflected the complex nature of the mammary tissue response to infection with *Staphylococcus* bacteria. The intricacy and complexity of gene expression patterns associated with *S. aureus* infections, both in blood leukocytes and milk somatic cells (MSCs), were emphasized by other researchers [15]. Moreover, the inflammation of mammary glands of other species, such as goat or pig exposed either to *Mycoplasma agalactiae* (goat) or to *E. coli* and *S. aureus* (pig), also resulted in many differentially expressed genes in the early response (until 24 h) to infection [21, 22].

### Functional classification of genes

Classifying DEGs according to their ontology, and so grouping them into similar categories in terms of gene function, identified a number of biological processes, molecular functions, and cellular components primarily involved in the immune response. In young cows infected with coagulase-negative *Staphylococci* (CoNS-1/2), increased expression levels of *CFB, CFH,* and *C4BP* were found, encoding complement system factors B and H as well as C4b-binding protein, respectively. In humans, these factors are an important part of the complement system acting against microbial infections [23] Similar results to ours were obtained by Rinaldi et al. [24] and Whelehan et al. [9]. These authors reported increased expression of acute phase proteins in mammary tissues infected with *E. coli* compared to bacteria free-tissues. Furthermore, the results of Whelehan et al. [9] showed elevated levels of *SAA3* gene transcripts (*SAA3* encodes components of serum amyloid A, belonging to acute phase proteins that act as opsonins) in the udder parenchyma of cows infected with *S. aureus*. In the present study, in CoPS-3/4 and CoNS-3/4 groups, as well as in CoPS-1/2 group, higher expression levels of genes encoding proteins of the canonical pathway of complement activation complex (*C6*, *C1S*, *C1QA*, *C1QB*, and *C4BP*) and the alternative route of the complement system (*CFB* and *CFH*) were observed. This is an interesting observation as the complement system is an example of cooperation between specific and non-specific immune mechanisms [25]. Activation of the complement pathway is important for a rapid response against pathogens, before an organism is able to develop specific mechanisms that operate slowly but more effectively. Numerous studies have reported activation by *S. aureus* of both alternative and classical complement pathways [26, 27], which is consistent with our results showing the activation of genes encoding proteins belonging to both pathways of complement action. Most of these genes, however, belong to the classical complement pathway, which could be due to the recurrent udder inflammation in the examined cows, resulting in production of specific antibodies activating the classical complement pathway. Two pathways of complement activation do not operate completely independently. The C3b component, generated by the classical pathway, can be deposited on the cell surface and thus may further initiate the formation of the alternative pathway. In the CoNS-1/2 group, the immune response to the pathogen relies on the activation of the alternative complement pathway. This may indicate that the organism of a young animal is able to combat infection without the activation of specific immune mechanisms, or that it meets with this certain type of bacteria for the first time.

In CoPS-1/2, CoPS-3/4, and CoNS-3/4 groups, four DEGs were identified with increased expression associated with antigen processing and presentation - *BOLA-DQB*, *BOLA-*DRB3, *BOLA-DQA2,* and *BOLA-DQA1*. These genes and the encoded proteins are critical for the immune system to clear infections [28]. Most studies confirm the paramount importance of class I and class II MHC molecules, which are key in resistance to infection not only in human [29] but also in goat and bovine, including resistance to mastitis [30–32]. Fitzpatrick et al. [33] showed MHC-II up-regulation in the connective tissue derived from *S. uberis* bacteria-infected cow udders. Swanson et al. [10] also reported a higher *HLA-DRA* (in human: HLA class II histocompatibility antigen, DR alpha chain) gene expression in the mammary tissues of cows after intramammary infection with *S. uberis* but not in healthy tissues. Moreover, Brand et al., [30] listed several genes belonging to the major histocompatibility complex (*HLA-DMA*, *HLA-DMB*, *HLA-DQA1*, *HLA-DQB1*, *HLA-DRA*, and *HLA-DRB1*) as a part of a “dendritic cell maturation” canonical pathway, which was affected by mastitic bacteria. According to Bonnefont et al. [34], the subunits of MHC class II, namely DQA1, DQA2, and DRB1, were also affected by *S. aureus*.

### Biochemical pathways

Two biochemical pathways were involved in the bovine immune response to infection with *Staphylococcus* bacteria: the Toll-like receptors pathway and the cytokine receptor interaction pathway. TLRs are membrane receptors that play a key role in the innate immune system, recognizing structurally conserved molecules derived from different microorganisms. Cells of the immune system which recognize the molecular patterns of a pathogen with TLRs, include macrophages and dendritic cells that respond to membrane components of Gram-positive and Gram-negative bacteria [35]. Stimulation of these cells by a pathogen through TLRs induces rapid activation of the innate immune mechanisms, manifested by increased synthesis of pro-inflammatory cytokines and activation of dendritic cells [36]. Toll-like receptors are located on the surface of macrophages, whose activation leads to the synthesis of TNF and IL-8 [12]. In our study, in the signaling pathway of Toll-like receptors, genes such as *TNF*, *IL-8*, *CXCL10* (Chemokine Ligand 10 – C-X-C Motif)), and *CD40* (TNF receptor superfamily) have been identified. This observation confirmed the results of other authors who have shown increased expression of *TNF*, *IL-8*, *CXCL10*, and *CD40* antigen genes in tissues of cow udders experimentally infected with *E. coli* and *S. aureus* [12, 37]. Moreover, Bonnefont et al. [34] stressed that the components of the chemokine signaling pathway are affected by *S. aureus* infection. The proteins encoded by *TNF*, *IL-8*, *CXCL10*, and CD40 genes (the same for both types of infections) are closely related to inflammation and are essential for the activation of neutrophils and their migration to sites of inflammation in the infected mammary gland. On the basis of our study, it can be concluded that bacterial infections by coagulase-positive and coagulase-negative *Staphylococci* evoke similar reactions in the innate immune system. This was also evidenced by the fact that the Toll-like receptors pathway leads to the activation of the next two pathways, which are also shared by both bacterial infections (CoPS and CoNS). One is the complement activation pathway, which involves the genes discussed above in relation to the process of acute inflammation. Another biochemical pathway involved in mammary gland response to bacterial pathogens is the cytokine receptor interaction pathway. In our study, in all groups of cows infected with *Staphylococci* bacteria, a higher level of expression was found for genes encoding chemokine receptors *CXCR4*, *CXCR6,* and *CX3CR1* (also known as the fractalkine receptor) as compared to the healthy groups of cows. Günther at el. [38], in a study on the secretory epithelial cells of bovine mammary glands infected with *E. coli,* showed an important role of the fractalkine receptor and other CXC receptors in the recruitment and migration of leukocytes to sites of inflammation. Additionally, results of studies on goat and porcine mammary gland epithelial cells also indicated on the influence of the infection on cytokine and chemokine expression levels [21, 22].

### Functional gene networks

Comparing CoPS-1/2 cows with controls, we identified a network of differentially expressed genes responsible for the migration of immune cells. In this network, *CD44*, *PLAU,* and *MMP7* genes can be distinguished, and they are strongly linked to other genes of the determined network. The *CD44* gene encodes a glycoprotein, the hyaluronain receptor, which is an important component of the extracellular matrix [39]. The interaction of CD44 with the glycoprotein hyaluronate (HA) has an important role in the adhesion of leukocytes to the vascular endothelium, as well as their migration to the sites of inflammation [40]. The results of more recent studies showed elevated *CD44* expression in inflammation of the cow mammary gland, particularly in the early stages of the inflammatory response [41]. The increased expression of *CD44* found in our study in a group of younger individuals (1st or 2nd lactations) may be indicative of the acute inflammation and tissue damage.

The *PLAU* gene encodes the urokinase plasminogen activator, which is a part of the fibrinolytic system. PLAU is a protease that converts plasminogen to plasmin by specific cleavage of the plasminogen protein [42]. The results of studies conducted by Lincoln and Leigh [43] and Moyes et al. [44] on a group of dairy cows infected with *S. uberis* also showed induction of the *PLAU* gene. The authors interpreted this result as an indicator of *S. uberis* virulence, causing inflammation of the mammary gland of cows. Plasmin catalyzes the degradation of fibrin and laminin to soluble peptides. Its activity is connected with elevated permeability of the epithelial barrier in the mammary gland during inflammation. The other function of plasmin is activation of MMP precursors [42]. Disintegration of these proteins is carried out either by direct action of plasmin, or indirectly through its activating effect on the MMP precursor cascade [45]. This corresponds to the results of our study, which also demonstrated the activation of the *MMP7* metalloproteinase gene, undergoing higher expression in the cows from the CoPS-1/2 group than in controls. Increased expression of the *MMP7* gene may result in the degradation of the extracellular matrix and the migration of immune cells to sites of inflammation.

In CoNS-3/4 and CoPS-3/4 groups, four and five significant intergenic networks were identified, respectively, which could be ascribed to two common key gene nodes: *TNF* and *FOS*. Furthermore, when comparing CoPS-1/2 cows with healthy ones, the *IL1-β* node (key) gene was identified. It exhibited, inseparably with *TNF*, activity in inflammation [5]. The *TNF*, *IL1-β,* and *FOS* genes belong to the Toll-like receptor and cytokine receptor interaction signaling pathways, and they were also identified in the CoPS-3/4 experimental group. The *FOS* gene encodes a transcription factor that is a component of the JUN/AP-1 trans-activating complex. In the basic state of a cell*, FOS* has a very low level of expression, but its transcript level increases within several minutes after stimulation by *S. uberis*; therefore, this gene is called “the early response gene” [43, 44]. It is involved in the regulation of cell proliferation, differentiation, and transformation [46]. Lützow et al. [12], after challenging bovine mammary tissues with *S. aureus*, showed increased expression of the *FOS* gene, which is involved in the TLR-2 and TLR-4 signaling pathway. In the studies of *S. uberis* infection, the *FOS* gene was shown to be a component of IL-6 and IL-10 signaling pathways [44].

AP1 has been reported play a crucial role in signaling pathways related to bacterial infections [47]. Functional AP1 sites have been identified in promoters of cytokine (interleukins, interferons) encoding genes [48–50] and other genes participating in the immune response. The C-JUN NH(2)-terminal kinase (JNK) regulates AP1 transcription factor activity and stimulates expression of pro-inflammatory mediators such as TNF [51]. The increased expression of the *FOS* gene found in our study indicated a strong stress caused by infection with coagulase-negative and coagulase-negative *Staphylococci*.

The *IL1B* gene encodes a protein that is a member of the cytokine – interleukin-1β family. This protein is produced by monocytes/macrophages and epithelial cells [5], whereas the *TNF-α* and *TNF-β* genes encode multifunctional pro-inflammatory cytokines that belong to the family of tumor necrosis factors (TNF) [52]. TNF-α plays an important role in the lactating cow’s mammary gland by mediating immune inflammatory responses in mastitis [53]. TNF mediates most pro-inflamatory effects that occur upon bacterial infection by promoting NFkB and MAPK activation [47]. Our identification of the proinflammatory cytokine genes as nodal (key) genes indicates their important role in the immune response induced by the two groups of bacteria studied (CoPS and CoNS). These results confirm those found in the literature, as the TNF-β and IL-1 cytokines were previously identified as the most important pro-inflammatory cytokines produced in inflammation. They are also critical in inflammation of the mammary gland of cows caused by *S. aureus* and *E. coli* [12, 54, 55]. As shown by Xiu et al. [56] other cytokines (e.g. Il-8; IL-1-alpha), including chemokines (chemokine ligand 5 – CXCL5; chemokine ligand 5 – C-C motif; chemokine receptor 7 – CXCL7) were upregulated after infection caused by *S. aureus*.

## Conclusion

Infection of cow mammary gland parenchyma with coagulase-negative or -positive *Staphylococci* affected genes, which encode proteins showing the same and/or similar molecular functions. Our results provided evidence that coagulase-positive *Staphylococci* caused a much stronger host response than coagulase-negative, resulting in increased number of DEGs as compared with uninfected tissues. Moreover, the response of the udder parenchyma to a *Staphyllococci* infection was stronger in older cows (lactations three or four) than was observed in younger ones (lactations one or two). Gene network analysis predicted DEGs associated with inflammation, cell migration, and the immune response to the infection site, as well as a humoral response in infections caused by both coagulase-positive and coagulase-negative groups of *Staphylococci*.

In both types of infections, caused either by coagulase-positive or coagulase-negative *Staphylococci*, a marked increase was observed in the expression levels of *FOS* and *TNF* genes, and genes encoding the major histocompatibility system proteins (*MHC)-BOLA-DQB*, *BOLA-DRB3*, or *BOLA-DQA1*. Therefore, we suggest that these particular genes play a key role in immune response synchronization of the cow’s udder secretory tissues to mastitis-causing bacteria.

Based on the results of microarray study, the following genes belonging to several physiological pathways (KEGG pathways) were selected for further studies: chemokine signaling pathway (*CCL2*, *CXCL5*, *HCK,CCR1*), cell adhesion molecules (CAMs) pathway (*BOLA-DQA2*, *BOLA-DQA1*, *F11R*, *ITGAL*, *CD86*), antigen processing and presentation pathway (*CD8A*, *PDIA3*, *LGMN*, *IFI30*, *HSPA1A*), and NOD-like receptor signaling pathway (*TNF*, *IL8*, *IL18*, *NFKBIA*). The listed genes are considered “main genes” involved in mammary gland defense, and these gene polymorphism study has already started to find the associations with resistance to mastitis aiming to include results to MAS in the future. Moreover, epigenetic studies have been conducted in parallel.

## Methods

### Animals and sample collection

The study was conducted on 30 Holstein-Friesian (HF) dairy cows of the Polish Black and White variety, between the first and fourth lactations. The animals were born and maintained in the Experimental Farm of Institute of Genetics and Animal Breeding in Jastrzębiec, Poland, and they were under constant veterinary supervision. The feeding and maintenance conditions of the animals were previously described [20]. Animals were culled at the third stage of lactation because of reproduction problems (without mastitis) or recurrent and incurable mammary gland infections caused by coagulase-positive or coagulase-negative *Staphylococci*. They were slaughtered in a registered slaughterhouse under constant monitoring conditions by the institutional authorities. The parenchyma samples were taken from deep in the secretory portion of the gland from each quarter immediately after slaughter. The authors were interested in the processes taking place only in parenchyma, therefore, the samples were rinsed in PBS to remove milk and blood from tissue samples and frozen in liquid nitrogen (120 samples total). Milk samples were taken from each quarter two days before slaughter and examined for the presence of bacteria. The exact methodology of the microbiological investigation was previously described [20] and is also shown in Additional file 11.

### Tissue samples

The samples were divided into five groups according to the lactation number and health status of the mammary gland, which was established on the basis of previous analysis of the presence of *Staphylococci* infection in quarter milk, somatic cell count (SCC) (IBC_M_, Bentley, USA), and lactose content (Fossomatic FT2, FOSS, Denmark). The control group (H) consisted of samples collected from healthy, pathogen-free mammary glands, and one sample was harvested from one cow. Three pathogen-free samples from each of the four lactations were taken for analysis (*N* = 12). The next four groups of samples were divided according to the type of pathogen bacteria and parity (cows with unfinished somatic development, being in the first and second lactations, or cows with complete somatic development, being in the third or fourth lactations). These four groups consisted of samples collected from cows with infections caused by coagulase-negative *Staphylococci*, being in their 1st or 2nd lactations or in the 3rd or 4^th^lactations (CoNS-1/2 and CoNS-3/4, respectively) and infected with coagulase-positive staphylococci in the 1st/2nd or 3rd/4th lactations (CoPS-1/2 and CoPS-3/4, respectively). Each group consisted of six samples derived from six different animals.

### RNA extraction, RNA pooling, sample preparation, and microarray hybridization scheme

The details of total RNA isolation from cow udder secretory tissues were described previously [20]. For each group, three independent RNA pools were obtained by mixing six individual RNA samples from bovine mammary glands, and one mg of pooled RNA was reverse transcribed using the Quick Amp Labeling Kit, Two-Color (Agilent, USA), according to the manufacturer’s protocol. For the arrays, combined RNA samples were hybridized in biological duplicates, and two technical replicates were performed for each hybridization. In both biological repetitions, the RNA samples were collected from the same individuals but from different udder quarters with the same health status. The total number of hybridizations was 16 (4 hybridizations for each of the treatment groups: CoPS-1/2, CoPS-3/4, CoNS-1/2, and CoNS-3/4). Hybridizations were performed to compare control RNA derived from healthy individuals, being in the first to fourth lactations (H-1/2/3/4). Established reference, control RNA represents standardized sample of physiologically “healthy” mammary gland RNA for the purpose of analysis and to help streamline and optimize of designed gene expression studies [57]. The broad representation of RNA in designed reference makes the control RNA useful to study of diseased mammary gland transcriptome regardless of the stage of lactation and the age of exanimated animals.

### RNA labeling, microarray hybridization, and fluorescent detection

Single strand cRNA was labeled with Cyanine 5-CTP or Cyanine 3-CTP (Agilent, USA). Reaction efficiency and activity were determined by NanoDrop. Hybridization to the Bovine (V2) Gene Expression Microarrays 4 × 44 K (Agilent, USA), washing, and scanning was performed according to the Two-Color Microarray-Based Gene Expression Analysis (Quick Amp Labeling) with Tecan HS Pro Hybridization. Following 17 h of hybridization, arrays were scanned on an Agilent G2565AA scanner. Images were quantified using Agilent Feature Extraction Software (version A.8.5.1.1). Pooling, labeling, and hybridization of RNA sample schemes in the microarray analysis are shown in Additional file 12.

### Microarray validation

Real-Time PCR (qPCR) was used to validate the microarray results. Ten genes that belonged to different functional clusters and differed in expression (*p* ≤ 0.05) between the control and CoNS-1/2, CoNS-3/4, CoPS-1/2, and CoPS-3/4 were selected for qPCR. These were: *HP*, *SAA3*, *CFB*, *CP*, *IL8*, *CA6*, *CA4*, *RYR3*, *ULBP3*, and *GPAM*. The real-time PCR validations were performed in triplicate of individual (unpooled) RNA samples.

Two reference genes were selected as the most stably expressed in the present experimental design, belonging to different functional classes from six housekeeping genes (HKGs) (Β-actin – *ACTB*, Glyceraldehyde-3P-dehydrogenase – *GAPDH*, Succinate dehydrogenase complex subunit A – *SDHA*, TATA box-binding protein – *TATABP*, Zeta polypeptide – *YWHAZ*, and Hypoxanthine phosphoribosyltransferase1 – *HPRT1*). The M-values of all putative reference genes were low and ranged between 0.6 and 0.3, and thus all met the criteria for proper references. However, *HPRT1* and *TBP* demonstrated the greatest stability in bovine mammary gland in the present experimental conditions and, therefore, were selected as references. The primer sequences, amplicon length, melting temperature, and GenBank accession numbers of both housekeeping and validated genes are summarized in Additional file 13.

Real-Time PCR amplification was performed with the Light Cycler 480 system (Roche, Germany) using 96-well optical plates with the SYBR Green technique. A PCR mix was prepared in a total volume of 20 μl: 10 μl water, 1 μl forward primer (10 μM), 1 μl reverse primer (10 μM), 2 μl cDNA, and 10 μl SYBR Green I Master Mix (2×) (Roche, Germany). The following amplification program was used: 5 min pre-incubation at 95 °C; 45 cycles amplification with 10 s at 95 °C for denaturation, 15 s at 58-60 °C for annealing, and 20 s at 72 °C for elongation. Negative controls (no cDNA) were run in the same reaction set. A dissociation stage was added to verify the presence of a gene specific peak and the absence of primer-dimer peaks. Real-time products were separated and assessed on 2% agarose gels.

Data normalization methods and selection of differently expressed genes are described in Additional file 14.

## Additional files

Additional file 1: Genes with up-regulated expression found in samples derived from cow mammary gland parenchyma infected with coagulase-positive *Staphylococci* in 1^st^or 2nd lactations (CoPS-1/2) vs. healthy cows (H). (XLS 86 kb)

Additional file 2: Genes with down-regulated expression found in samples derived from cow mammary gland parenchyma infected with coagulase-positive *Staphylococci* in 1st or 2nd lactations (CoPS-1/2) vs. healthy cows (H). (XLS 33 kb)

Additional file 3: Figure S1. **Figure S1.** Significantly enriched Gene Ontology (GO) categories of genes differentially expressed in mammary secretory tissue between CoPS-1/2 and H cows. **Figure S2.** Gene network graphical representation. Cellular movement, hematological system development and function, and immune cell trafficking in secretory tissue between CoPS-1/2 and H cows. **Figure S3**. Significantly enriched Gene Ontology (GO) categories of genes differentially expressed in mammary secretory tissue between CoPS-3/4 and H cows. **Figure S4.** Gene network graphical representation: (A) Metabolic Disease, (B) Cellular Assembly and Organization, (C) Inflammatory Response, (D) Infectious Disease, Tissue Development in secretory tissue between CoPS-3/4 and H cows. **Figure S5. **Significantly enriched Gene Ontology (GO) categories of genes differentially expressed in mammary secretory tissue between CoNS-1/2 and H cows. **Figure S6.** Gene network graphical representation. Cell-To-Cell Signaling and Interaction in mammary secretory tissue between CoNS-1/2  and H cows. **Figure S7.** Significantly enriched Gene Ontology (GO) categories of genes differentially expressed in mammary secretory tissue between CoNS- 3/4 and H cows. **Figure S8.** Gene network graphical representation: (A) Cell Morphology, (B) Cell Assembly and Organization, (C) Cellular Growth and Proliferation, (D) Organismal Injury and Abnormalities, (E) Humoral Immune Response - in secretory tissue between CoNS-3/4 and H cows.(DOCX 1683 kb)

Additional file 4: Genes with up-regulated expression found in samples derived from cow mammary gland parenchyma infected with coagulase-positive *Staphylococci* in 3rd or 4th lactations (CoPS-3/4). (XLS 107 kb)

Additional file 5: Genes with down-regulated expression found in samples derived from cow mammary gland parenchyma infected with coagulase-positive *Staphylococci* in 3rd or 4th lactations (CoPS-3/4). (XLS 42 kb)

Additional file 6: Genes with up-regulated expression found in samples derived from cow mammary gland parenchyma infected with coagulase-negative *Staphylococci* in 1st or 2nd lactations (CoNS-1/2). (XLS 21 kb)

Additional file 7: Genes with down-regulated expression found in samples derived from cow mammary gland parenchyma infected with coagulase-negative *Staphylococci* in 1st or 2nd lactations (CoNS-1/2). (XLS 20 kb)

Additional file 8: Genes with up-regulated expression found in samples derived from cow mammary gland parenchyma infected with coagulase-negative *Staphylococci* in 3rd or 4th lactations (CoNS-3/4). (XLS 81 kb)

Additional file 9: Genes with down-regulated expression found in samples derived from cow mammary gland parenchyma infected with coagulase-negative *Staphylococci* in 3rd or 4th lactations (CoNS-3/4). (XLS 29 kb)

Additional file 10: Table S1. Gene clusters differing in expression between the CoPS-1/2 (coagulase-positive *Staphyloccoci* in 1st or 2nd lactation) and H (Healthy) groups in the parenchyma of the cow mammary gland. **Table S2.** Gene clusters differing in expression between the CoPS-3/4 (coagulase-positive *Staphyloccoci* in 3rd or 4th lactation) and H (Healthy) groups in the parenchyma of the cow mammary gland. **Table S3.** Gene clusters differing in expression between the CoNS-1/2 (coagulase-negative *Staphyloccoci* in 1st or 2nd lactation) and H (Healthy) groups in the parenchyma of the cow mammary gland. **Table S4.** Gene clusters differing in expression between the CoNS-3/4 (coagulase-positive *Staphyloccoci* in 3rd or 4th lactation) and H (Healthy) groups in the parenchyma of the cowmammary gland. (DOCX 65 kb)

Additional file 11: Microbiological examination. (DOCX 13 kb)

Additional file 12: Pooling, labeling, and hybridization of RNA sample schemes used in microarray analysis. (DOCX 680 kb)

Additional file 13: Table S5. The primer sequences, amplicon length, melting temperature and no. of GenBank access of housekeeping genes examined to use in qPCR analysis. **Table S6.** The primer sequences, amplicon length, melting temperature and No. of GenBank access of validated genes. (DOCX 21 kb)

Additional file 14: Data normalization and selection of differently expressed genes (DEGs) (DOCX 15 kb)

## Abbreviations

ANXA2: Annexin A2
BoLA: Bovine lymphocyte antigen
BOLA-DQA1: Major histocompatibility complex, class II, DQ alpha, type 1 [*Bos taurus* (cattle)]
BOLA-DQA2: Major histocompatibility complex, class II, DQ alpha 2 [*Bos taurus* (cattle)]
BOLA-DQB: Major histocompatibility complex, class II, DQ beta [*Bos taurus* (cattle)]
BOLA-DRB3: Major histocompatibility complex, class II, DRB3 [*Bos taurus* (cattle)]
C1QA: Complement component 1, q subcomponent, A chain
C1QB: Complement component 1, q subcomponent, B chain
C1S: Complement component 1, s subcomponent
C3B: Complement Component 3b
C4BP: Complement component 4 binding protein
C6: Complement Component 6
CA4: Carbonic anhydrase 4
CA6: Carbonic anhydrase 6
CAMs: Cell adhesion molecules
CCL26: Chemokine (C-C motif) ligand 2
CD40: CD40 molecule
TNF: receptor superfamily
CD44: Cell-surface glycoprotein
CFB: complement factor B
CFH: Complement factor H
COL1A1: Collagen, type I, alpha 1
CoNS-1/2 group: Cows infected with coagulase-negative *Staphylococci*, being in their 1st and 2nd lactations
CoNS-3/4: Cows infected with coagulase-negative staphylococci, being in their 3rd and 4th lactation
CoPS-1/2 group: Cows infected with coagulase-positive *Staphylococci*, in 1st or 2nd lactation
CoPS-3/4 group: Cows infected with coagulase-positive Staphylococci, in 3rd or 4th lactation
COX3: Cytochrome c oxidase subunit 3
CP: Cytoplasmic polyadenylation
CX3CR1: Fractalkine receptor
CXCL10: Chemokine (C-X-C Motif) Ligand 10
CXCR4: C-X-C chemokine receptor type 4 also known as fusin or CD184
CXCR6: C-X-C chemokine receptor type 6
DEGs: Differentially expressed genes
EGR1: Early growth response protein 1
FOS: *Bos taurus* FBJ murine osteosarcoma viral oncogene homolog
FST: Follistatin
GPAM: Glycerol-3-phosphate acyltransferase
GPD1: Glycerolphosphate Dehydrogenase 1
H: Healthy cows
HLA: human lymphocyte antigen
HLA-DRA: HLA class II histocompatibility antigen, DR alpha chain
HP: Haptoglobin
IL-10: Interleukin-10
IL1B: Cytokine-interleukin-1β
IL-2: Interleukin-2
IL2R: Interleukin-2 receptor
IL-6: interleukin-6
IL-8: Interleukin 8
ILB: Interleukin beta
JUN/AP-1: JUN/AP-1 trans-activating complex
KEGG: Kyoto Encyclopedia of Genes and Genomes
LPS: Lipopolysaccharide
MHC: Proteins of major histocompatibility complex
MMP7: Matrix Metallopeptidase 7
MSCs: Milk somatic cells
PLAU: Plasminogen activator, urokinase
PTGS2: Prostaglandin endoperoxide synthase-2
qPCR: Real-time quantitative PCR
RYR3: Ryanodine receptor type 3
SAA1: Serum amyloid A1
SAA3: Serum amyloid A3
SaS: Staphylococcal culture
SCD: Stearoyl-CoA desaturase
SOD2: Superoxide dismutase 2
TGFB3: Transforming growth factor beta 3
TGF-β: Transforming growth factor beta
Th: T helper cells
TIMP1: TIMP metallopeptidase inhibitor 1
TLR-2: Toll-like receptor 2
TLR-4: Toll-like receptor 4
TNF: Tumor Necrosis Factor
UBC: Ubiquitin
ULBP3: UL16 binding protein 3
