# Supplementary material for: Transcriptome profiling of Staphylococci-infected cow mammary gland parenchyma
Source: BMC Vet Res. 2017 Jun 6;13:161. doi: 10.1186/s12917-017-1088-2 (PMC5477815; doi:10.1186/s12917-017-1088-2)
Supplement: Supplementary file 3 — Figure S1. Significantly enriched Gene Ontology (GO) categories of genes differentially expressed in mammary secretory tissue between CoPS-1/2 and H cows. Figure S2. Gene network graphical representation. Cellular movement, hematological system development and function, and immune cell trafficking in secretory tissue between CoPS-1/2 and H cows. Figure S3. Significantly enriched Gene Ontology (GO) categories of genes differentially expressed in mammary secretory tissue between CoPS-3/4 and H cows. Figure S4. Gene network graphical representation: (A) Metabolic Disease, (B) Cellular Assembly and Organization, (C) Inflammatory Response, (D) Infectious Disease, Tissue Development in secretory tissue between CoPS-3/4 and H cows. Figure S5. Significantly enriched Gene Ontology (GO) categories of genes differentially expressed in mammary secretory tissue between CoNS-1/2 and H cows. Figure S6. Gene network graphical representation. Cell-To-Cell Signaling and Interaction in mammary secretory tissue between CoNS-1/2 and H cows. Figure S7. Significantly enriched Gene Ontology (GO) categories of genes differentially expressed in mammary secretory tissue between CoNS- 3/4 and H cows. Figure S8. Gene network graphical representation: (A) Cell Morphology, (B) Cell Assembly and Organization, (C) Cellular Growth and Proliferation, (D) Organismal Injury and Abnormalities, (E) Humoral Immune Response - in secretory tissue between CoNS-3/4 and H cows.(DOCX 1683 kb) [file 12917_2017_1088_MOESM3_ESM.docx]

**Additional file 3**

**A)**

**biological process**


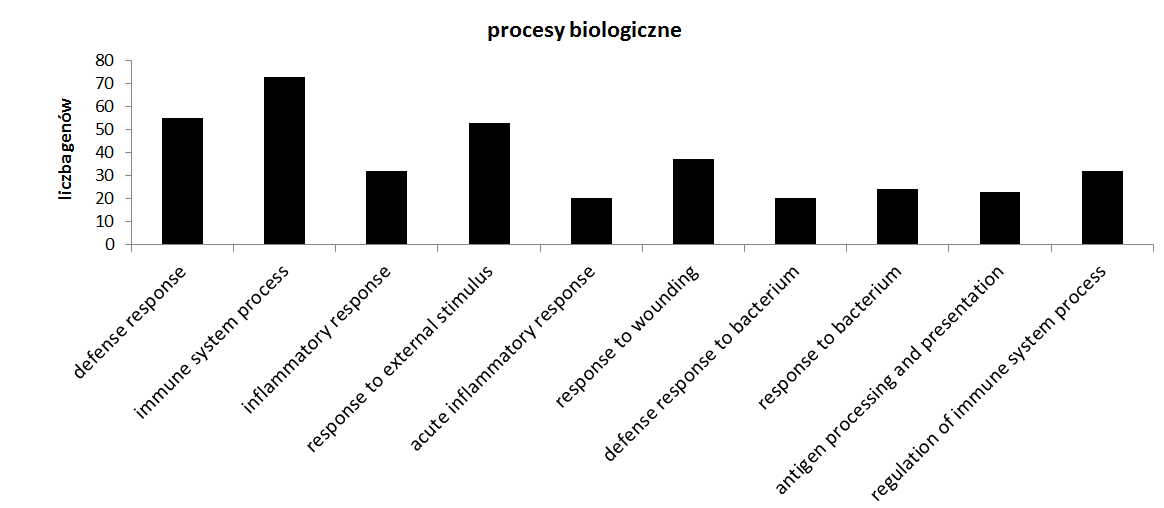


**number of genes**

**B)**


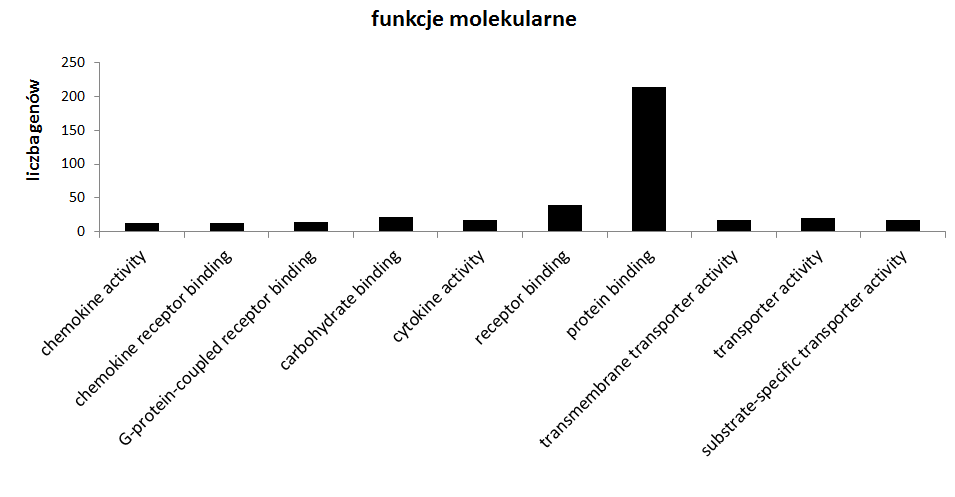


**molecular function**

**number of genes**

**C)**


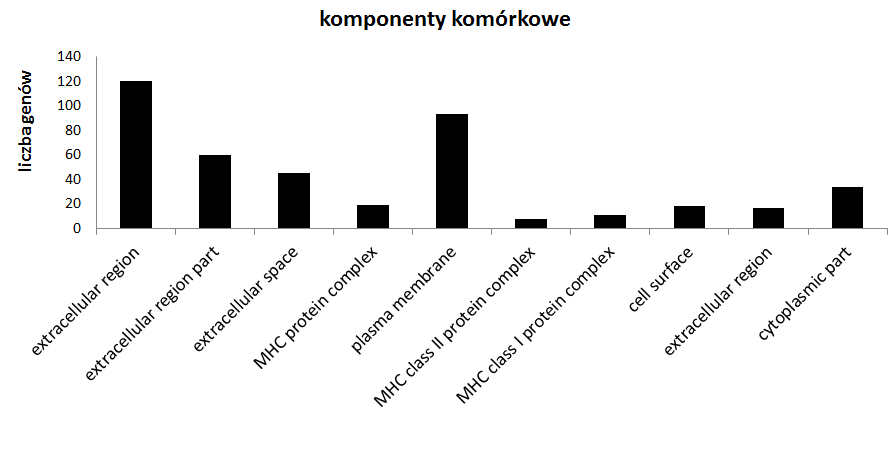


**number of genes**

**cellular component**

Figure S1. Significantly enriched Gene Ontology (GO) categories of genes differentially expressed in secretory tissue between CoPS-1,2 and H cows. (A) GO biological process terms, (B) GO molecular function terms, (C) GO cellular component terms (there are shown 10 categories of cellular components of the most differentially expressed genes, according to the increasing P value).


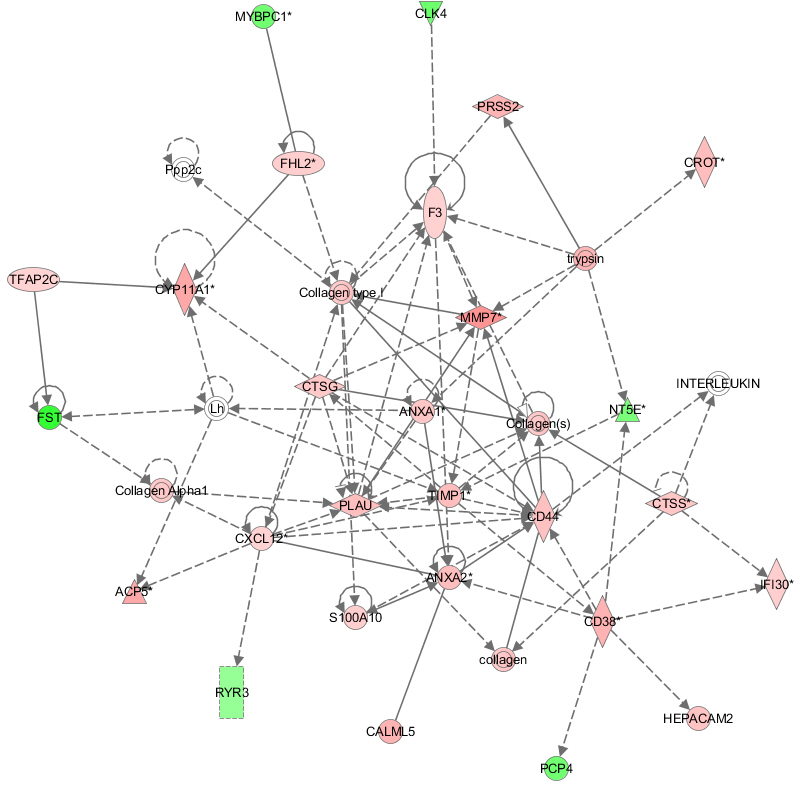


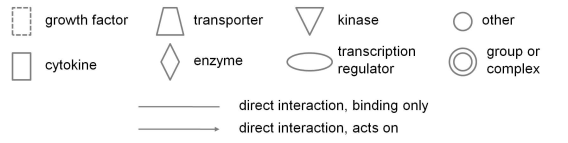


Figure S2**.** Gene network graphical representation. *Cellular movement, hematological system development and function, immune cell trafficking* - in secretory tissue between CoPS-1,2 and H cows.Genes or genes' products are represented as nodes, and biological relationship between two nodes is represented as an edge (line). The node color indicates genes up-regulated in infected cows (red) or up-regulated in healthy cows (green). Nodes are displayed using various shapes that represent the functional class of a gene product.

**A)**


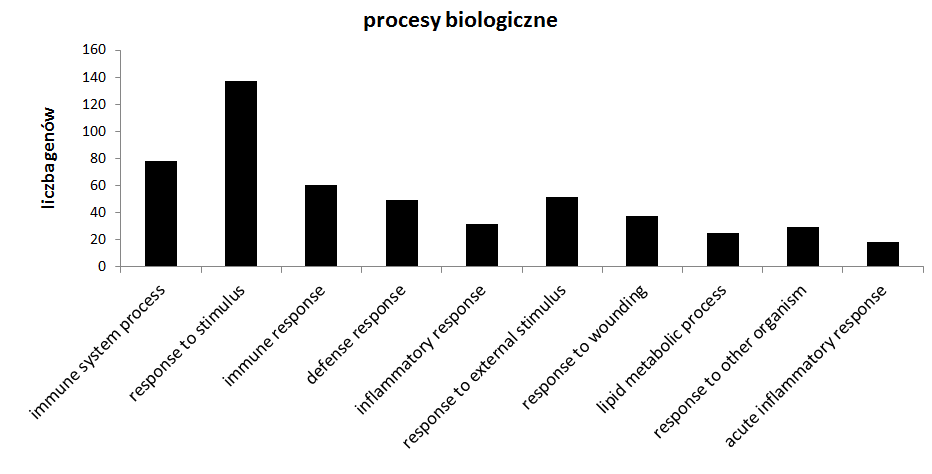


**number of genes**

**biological process**

**B)**


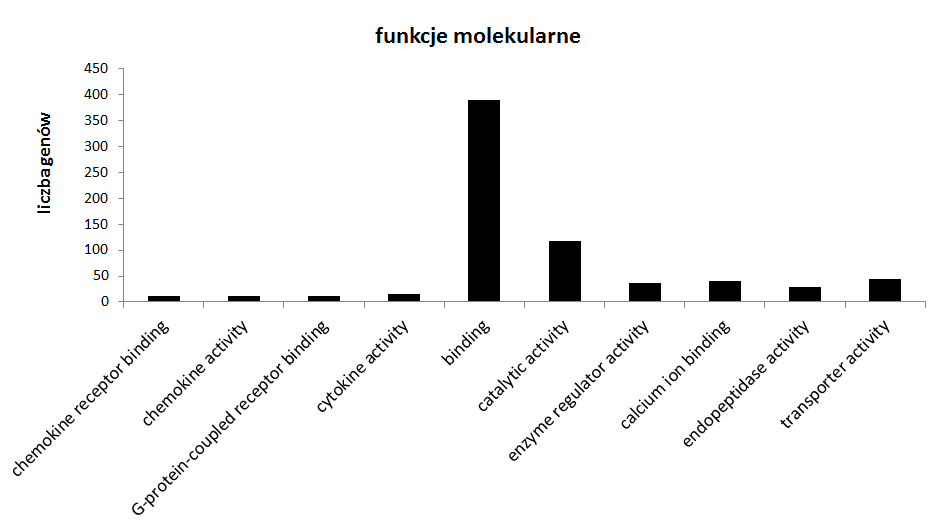


**molecular function**

**number of genes**

**C)**


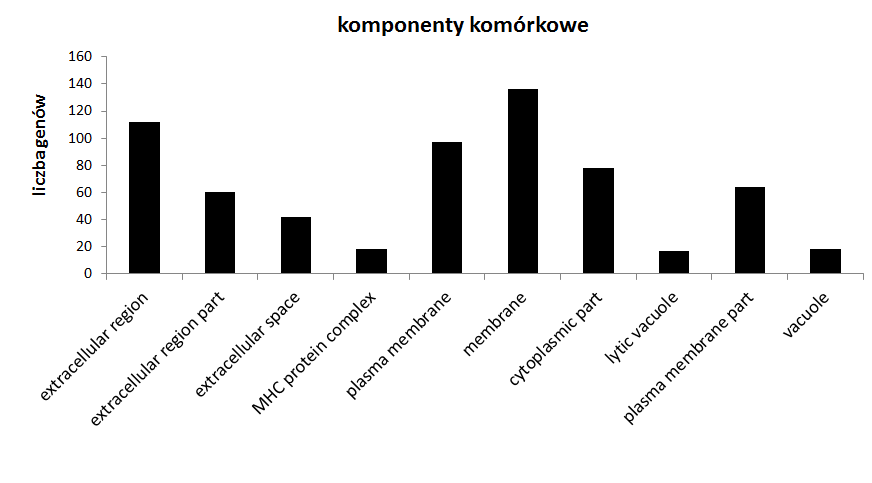


**cellular component**

**number of genes**

Figure S3. Significantly enriched Gene Ontology (GO) categories of genes differentially expressed in secretory tissue between CoPS-3,4 and H cows. (A) GO biological process terms, (B) GO molecular function terms, (C) GO cellular component terms (there are shown 10 categories of cellular components of the most differentially expressed genes, according to the increasing P value).

**A**


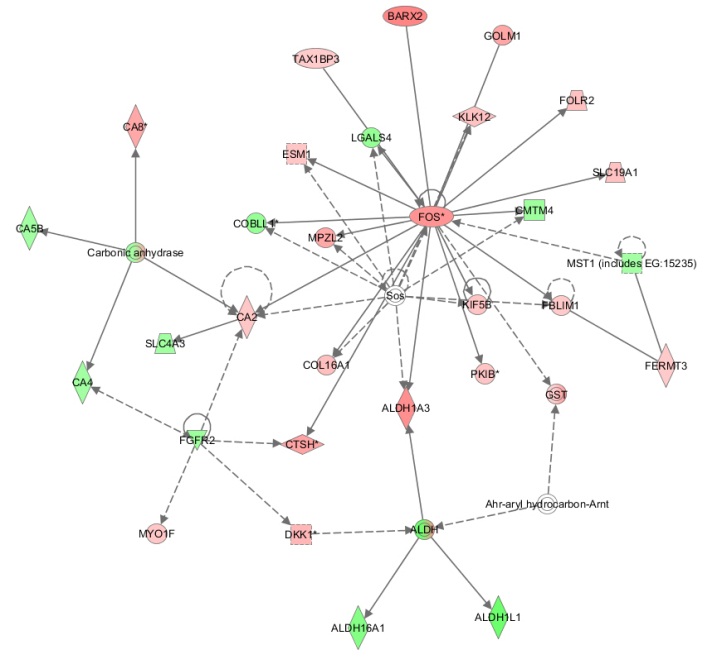


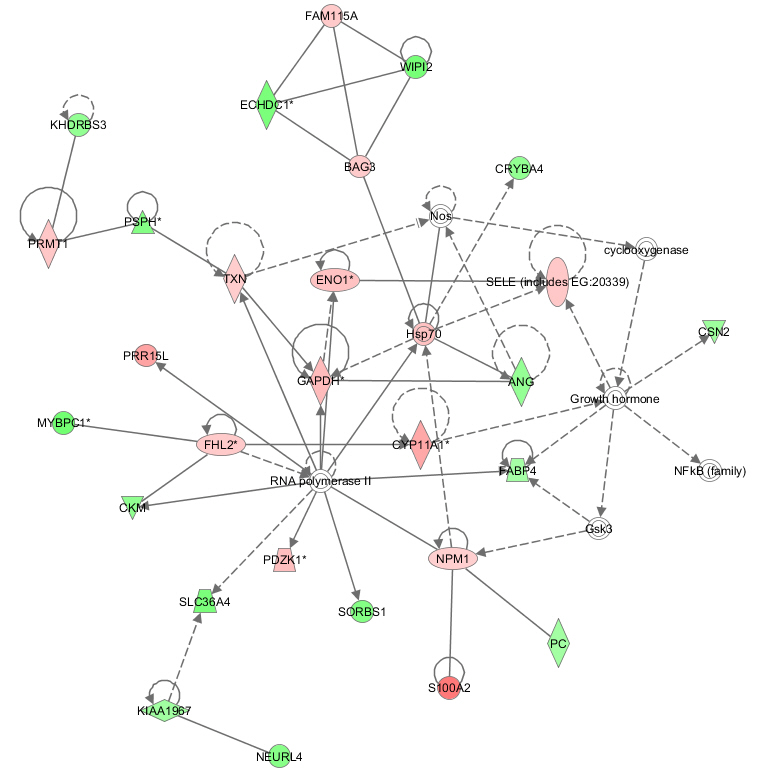


**B**


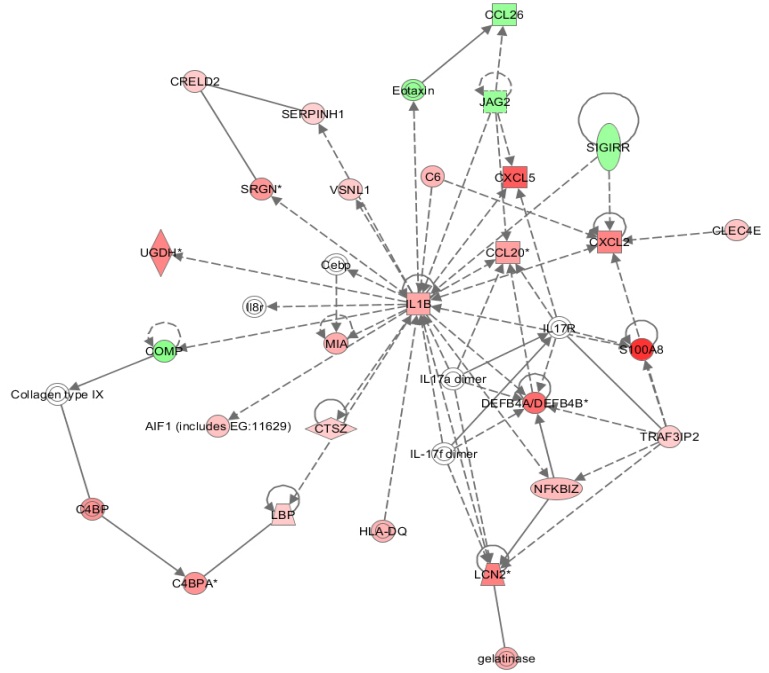


**C**


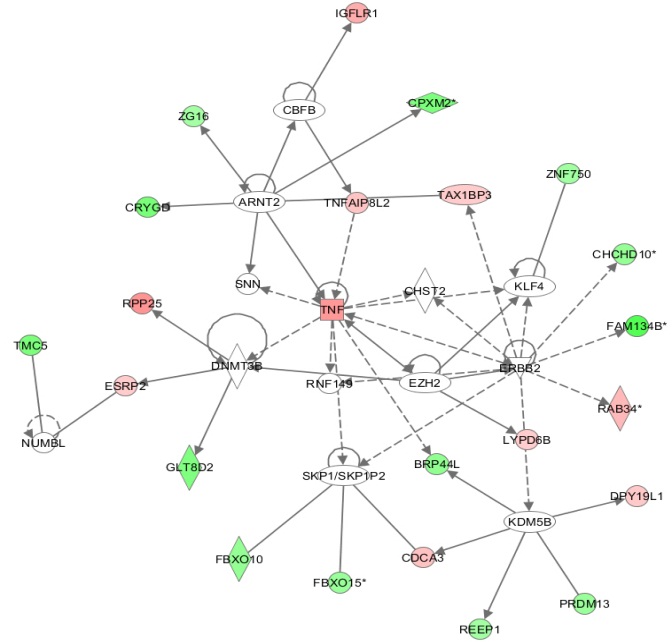


**D**


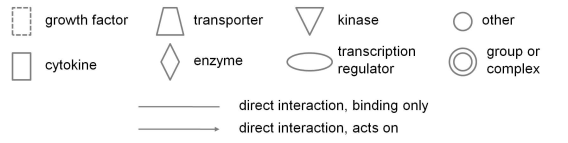


Figure S4.Gene network graphical representation: (**A**) *Metabolic Disease*, (**B**) *Cellular Assembly and Organization*, (**C**) *Inflammatory Response*, (**D**) *Infectious Disease, Tissue Development.* In secretory tissue between CoPS-3,4 and H cows Genes or genes' products are represented as nodes, and biological relationship between two nodes is represented as an edge (line). The node color indicates genes up-regulated in infected cows (red) or up-regulated in healthy cows (green). Nodes are displayed using various shapes that represent the functional class of a gene product.

**A)**

**
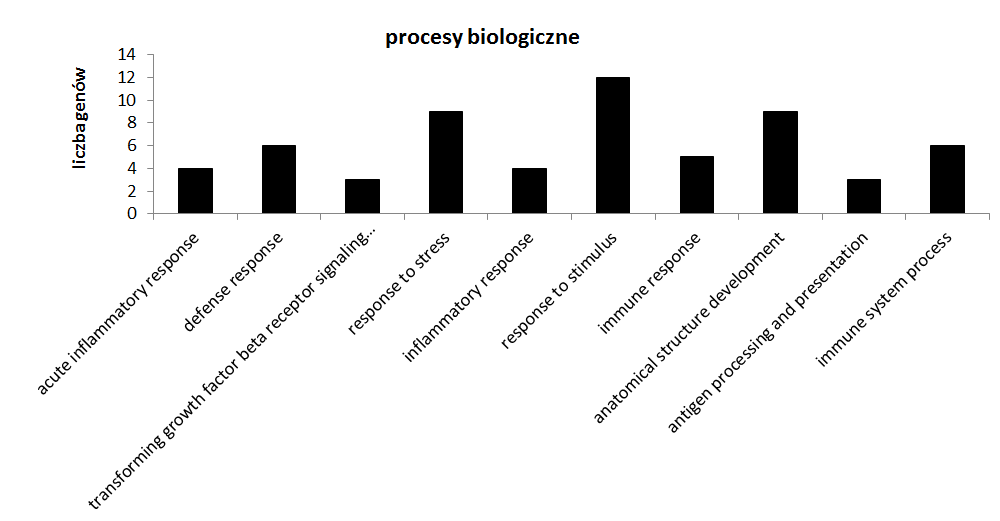
**

**number of genes**

**biological process**

**B)**

**
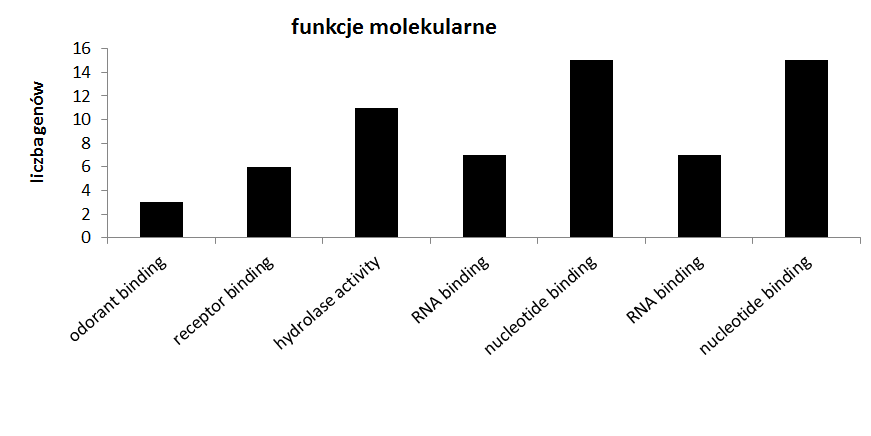
**

**number of genes**

**molecular function**

**C)**

**
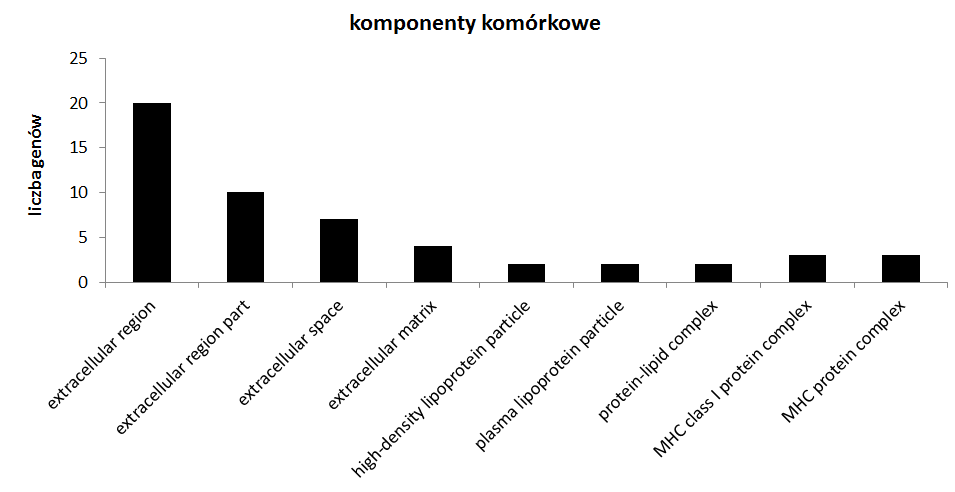
**

**number of genes**

**cellular component**

Figure S5. Significantly enriched Gene Ontology (GO) categories of genes differentially expressed in secretory tissue between CoNS-1,2 and H cows. (A) GO biological process terms, (B) GO molecular function terms, (C) GO cellular component terms (there are shown the categories of cellular components of the most differentially expressed genes, according to the increasing P value).

**
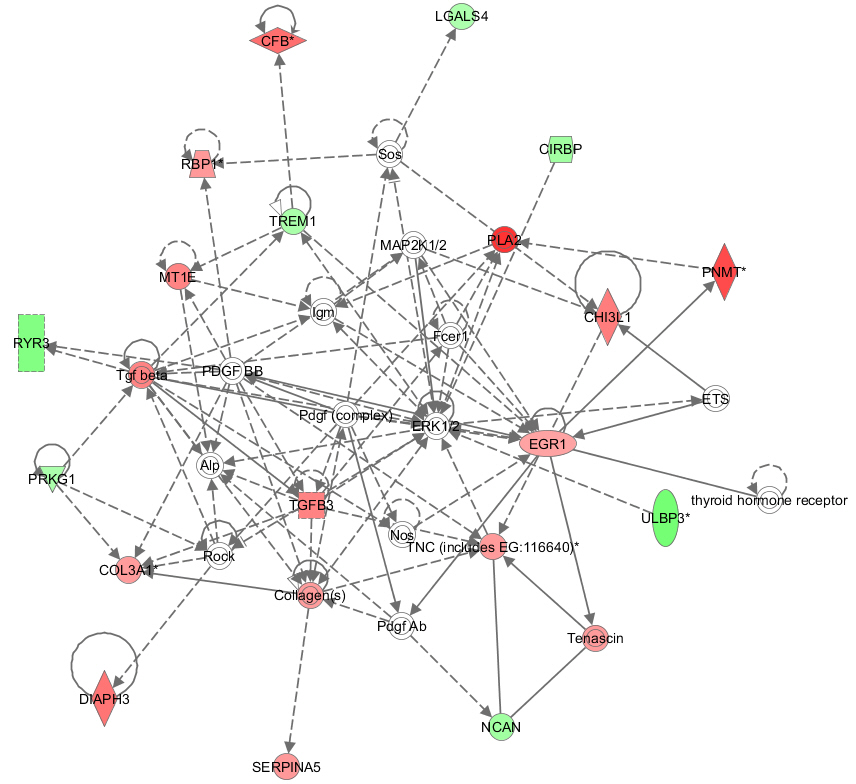
**


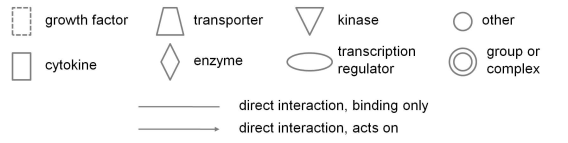


Figure S6. Gene network graphical representation .*Cell-To-Cell Signaling and Interaction* in secretory tissue between CoNS-1,2 and H cows. Genes or genes' products are represented as nodes, and biological relationship between two nodes is represented as an edge (line). The node colour indicates genes up-regulated in infected cows (red) or up-regulated in healthy cows (green). Nodes are displayed using various shapes that represent the functional class of a gene product.

**A)**

**B)
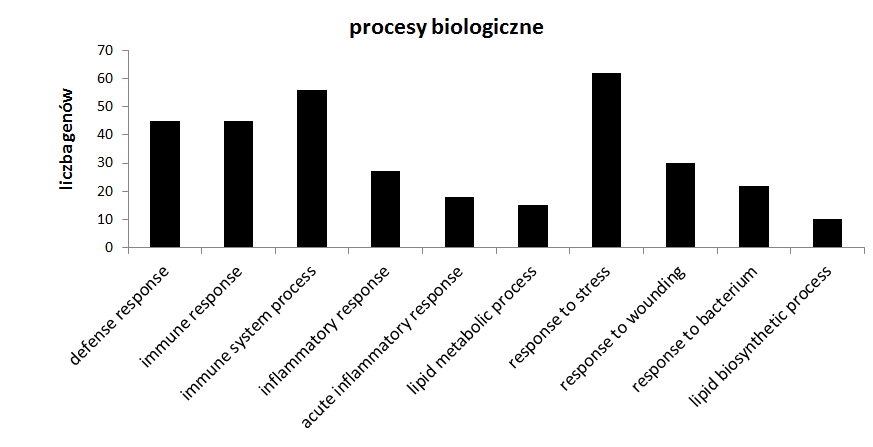
**

**number of genes**

**biological process**

**
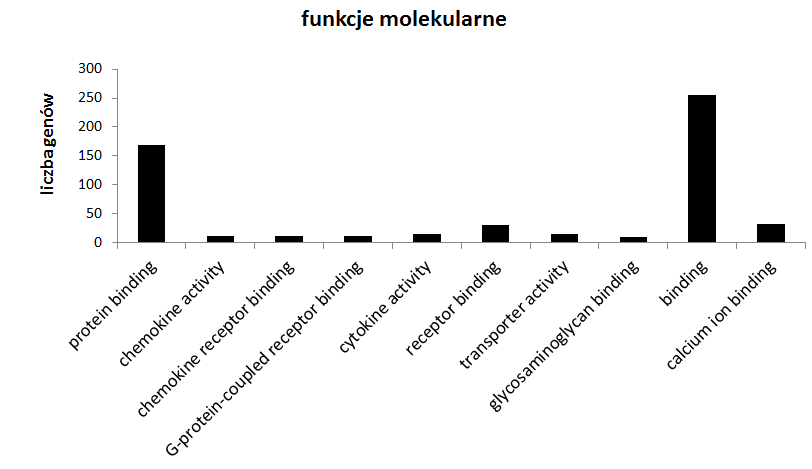
**

**molecular function**

**number of genes**

**C)**


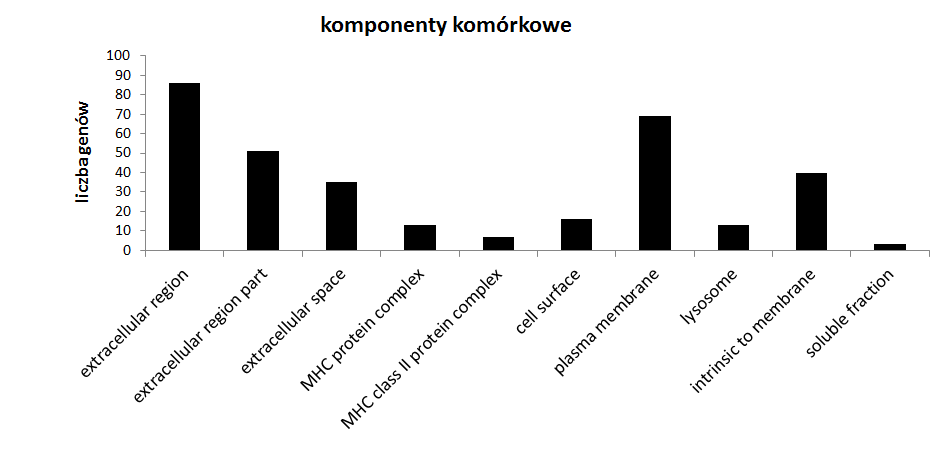


**cellular component**

**number of genes**

Figure S7. Significantly enriched Gene Ontology (GO) categories of genes differentially expressed in secretory tissue between CoNS- 3,4 and H cows. (A) GO biological process terms, (B) GO molecular function terms, (C) GO cellular component terms. (there are shown 10 categories of cellular components of the most differentially expressed genes, according to the increasing P value).


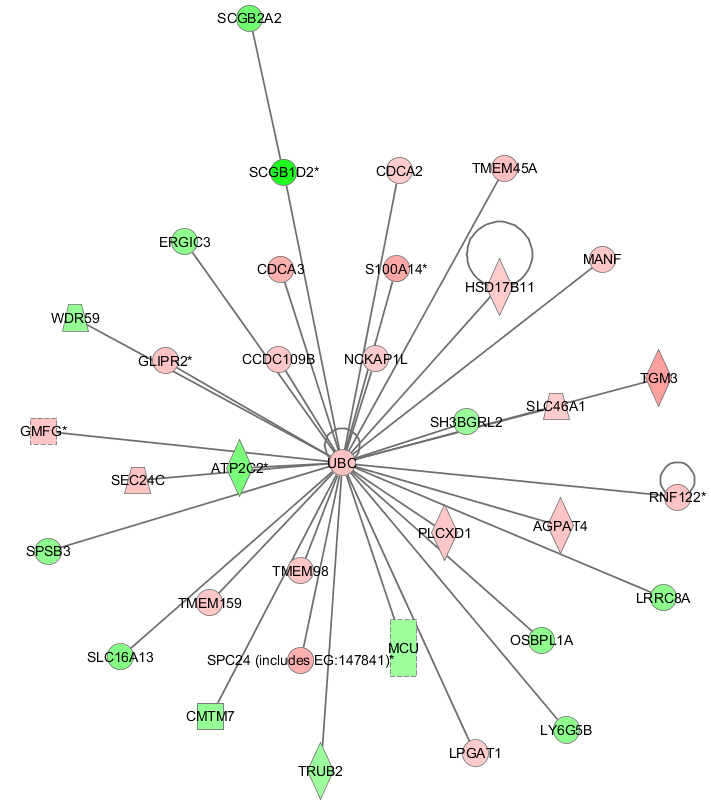


**A**

**B**


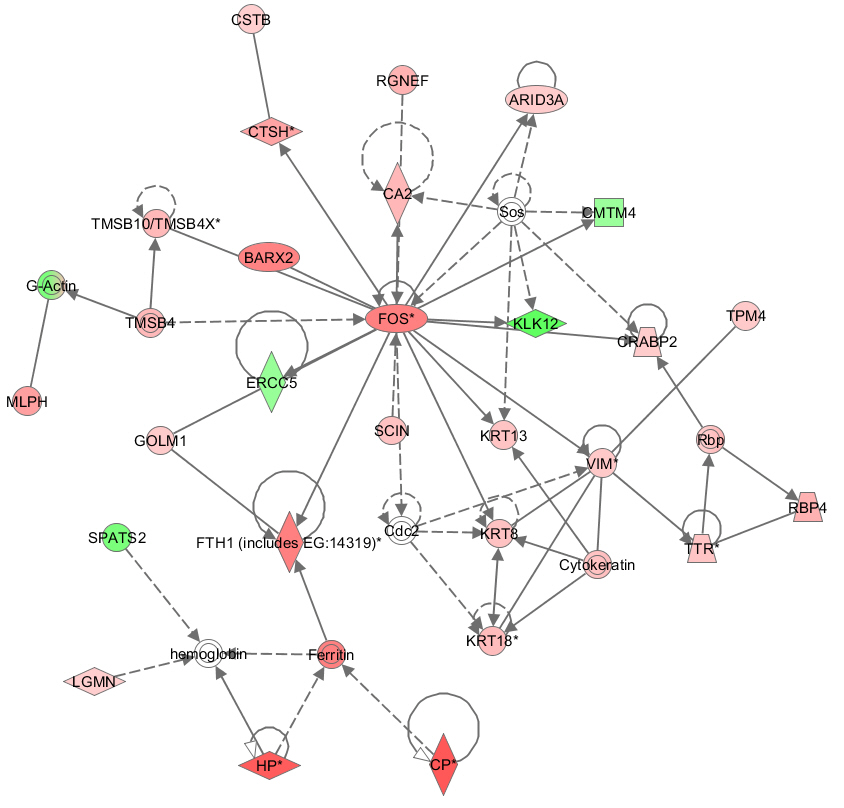


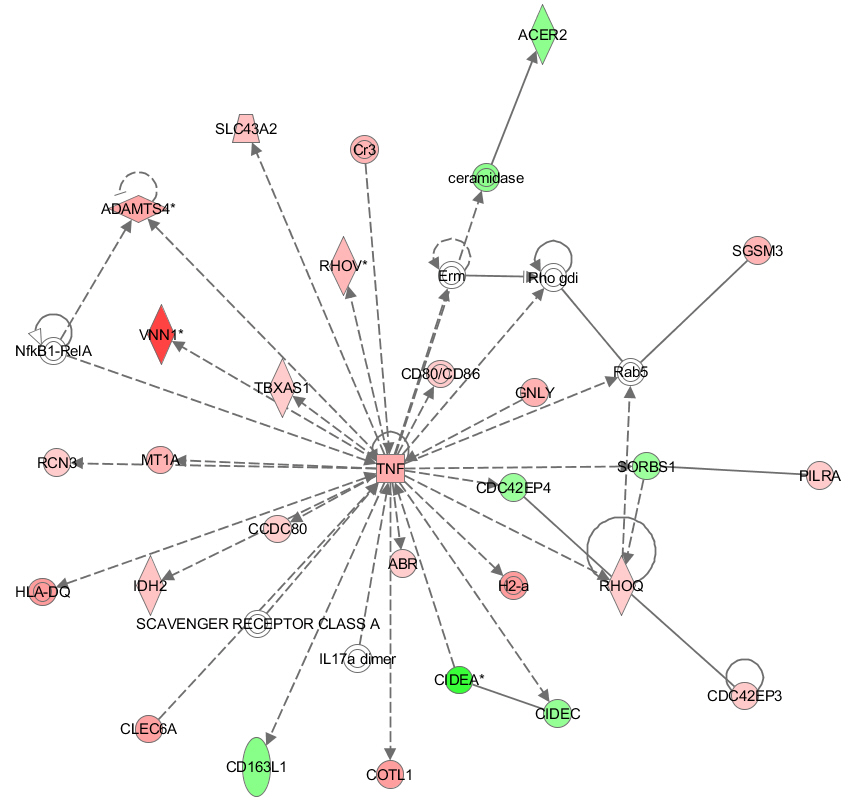


**C**


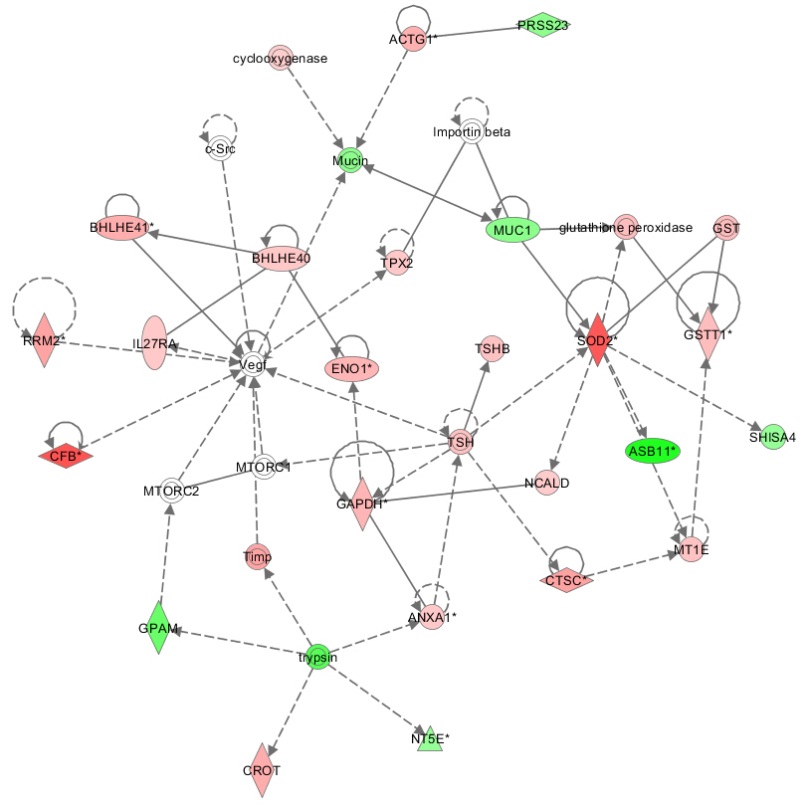


**D**


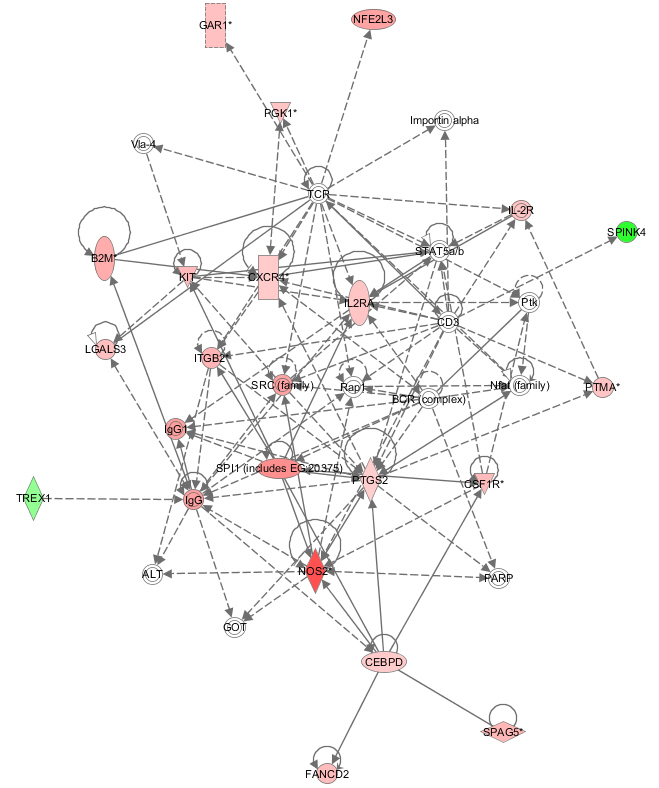


**E**


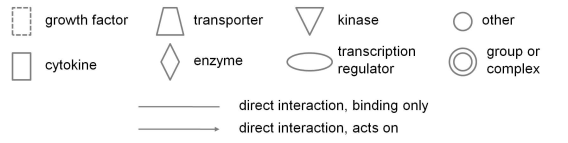


Figure S8. Gene network graphical representation.(**A**) *Cell Morphology*, (**B**) *Cell Assembly and Organization*, (**C**) *Cellular Growth and Proliferation*, (**D**) *Organismal Injury and Abnormalities*, (**E**) *Humoral Immune Response* - in secretory tissue between CoNS-3,4 and H cows Genes or genes' products are represented as nodes, and biological relationship between two nodes is represented as an edge (line). The node color indicates genes up-regulated in infected cows (red) or up-regulated in healthy cows (green). Nodes are displayed using various shapes that represent the functional class of a gene product.
