# Supplementary material for: Transcriptome profiling of Staphylococci-infected cow mammary gland parenchyma
Source: BMC Vet Res. 2017 Jun 6;13:161. doi: 10.1186/s12917-017-1088-2 (PMC5477815; doi:10.1186/s12917-017-1088-2)
Supplement: Supplementary file 10 — Gene clusters differing in expression between the CoPS-1/2 (coagulase-positive Staphyloccoci in 1st or 2nd lactation) and H (Healthy) groups in the parenchyma of the cow mammary gland. Table S2. Gene clusters differing in expression between the CoPS-3/4 (coagulase-positive Staphyloccoci in 3rd or 4th lactation) and H (Healthy) groups in the parenchyma of the cow mammary gland. Table S3. Gene clusters differing in expression between the CoNS-1/2 (coagulase-negative Staphyloccoci in 1st or 2nd lactation) and H (Healthy) groups in the parenchyma of the cow mammary gland. Table S4. Gene clusters differing in expression between the CoNS-3/4 (coagulase-positive Staphyloccoci in 3rd or 4th lactation) and H (Healthy) groups in the parenchyma of the cowmammary gland. (DOCX 65 kb) [file 12917_2017_1088_MOESM10_ESM.docx]

**Additional file 10**

**Table S1**. Gene cluster differing in expression between the CoPS-1/2 (coagulase-positive *Staphyloccoci* in 1^st^ and 2^nd^ lactation)and H (Healthy) groups in the parenchyma of dairy cattle mammary gland.

Cluster 1 Number of genes p

GO:0006952~defense response 57 0.025

GO:0042742~defense response to bacterium 21 0.061

GO:0009617~response to bacterium 25 0.053

Cluster 2

GO:0019882~antigen processing and presentation 25 0.010

GO:0042611~MHC protein complex 21 0.044

GO:0042612~MHC class I protein complex 13 0.013

Cluster 3

GO:0006935~chemotaxis 18 0.023

GO:0008009~chemokine activity 13 0.056

GO:0042379~chemokine receptor binding 13 0.056

GO:0005125~cytokine activity 17 0.017

Cluster 4

GO:0006952~defense response 57 0.025

GO:0006954~inflammatory response 33 0.068

GO:0002526~acute inflammatory response 21 0.022

GO:0009611~response to wounding 38 0.015

GO:0002684~positive regulation of immune system process 27 0.017

GO:0006959~humoral immune response 11 0.072

GO:0006956~complement activation 9 0.043

GO:0002541~activation of plasma proteins involved in acute inflammatory response 9 0.043

GO:0048584~positive regulation of response to stimulus 21 0.051

GO:0002252~immune effector proces 16 0.063

GO:0002250~adaptive immune response 12 0.088

GO:0045087~innate immune response 14 0.028

GO:0002443~leukocyte mediated immunity 12 0.030

GO:0016064~immunoglobulin mediated immune response 10 0.048

GO:0002449~lymphocyte mediated immunity 11 0.056

GO:0019724~B cell mediated immunity 10 0.063

GO:0050778~positive regulation of immune response 15 0.089

GO:0051605~protein maturation by peptide bond cleavage 10 0.021

GO:0016485~protein processing 11 0.056

GO:0002455~humoral immune response mediated by circulating immunoglobulin 6 0.074

GO:0006958~complement activation. classical pathway 6 0.074

GO:0051604~protein maturation 11 0.090

GO:0002253~activation of immune response 10 0.001

Cluster 5

GO:0048002~antigen processing and presentation of peptide antigen 11 0.018

GO:0002504~antigen processing and presentation of peptide or polysaccharide antigen via MHCclass II 8 0.021

GO:0002478~antigen processing and presentation of exogenous peptide antigen 5 0.003

GO:0019884~antigen processing and presentation of exogenous antigen 5 0.008

GO:0019886~antigen processing and presentation of exogenous peptide antigen via MHC classII 4 0.012

GO:0002495~antigen processing and presentation of peptide antigen via MHC class II 4 0.012

Cluster 6

GO:0050900~leukocyte migration 10 0.010

GO:0030595~leukocyte chemotaxis 6 0.001

GO:0060326~cell chemotaxis 6 0.001

GO:0007159~leukocyte adhesion 5 0.002

GO:0016477~cell migration 15 0.005

GO:0030593~neutrophil chemotaxis 4 0.005

GO:0006928~cell motion 17 0.009

GO:0048870~cell motility 15 0.010

GO:0051674~localization of cel 15 0.010

**TableS2.** Gene cluster differing in expression between the CoPS-3/4 (coagulase-positive *Staphyloccoci* in 3^rd^ and 4^th^ lactation) and H (Healthy) groups in the parenchyma of dairy cattle mammary gland.

Cluster 1 Number of genes p

GO:0006952~defense response 57 0.036

GO:0006954~inflammatory response 35 0.002

GO:0009611~response to wounding 44 0.005

GO:0002526~acute inflammatory response 20 0.003

Cluster 2

GO:0019882~antigen processing and presentation 23 0.005

GO:0048002~antigen processing and presentation of peptide antigen 12 0.002

GO:0042611~MHC protein complex 18 0.009

GO:0002474~antigen processing and presentation of peptide antigen via MHC class I 8 0.004

GO:0042612~MHC class I protein complex 11 0.004

Cluster 3

GO:0016477~cell migration 24 0.006

GO:0006928~cell motion 28 0.007

GO:0051674~localization of cel 25 0.008

GO:0048870~cell motility 25 0.008

Cluster 4

GO:0008610~lipid biosynthetic proces 32 0.001

GO:0046394~carboxylic acid biosynthetic process 21 0.001

GO:0016053~organic acid biosynthetic process 21 0.001

GO:0006633~fatty acid biosynthetic process 15 0.003

GO:0006631~fatty acid metabolic process 20 0.001

Cluster 5

GO:0002526~acute inflammatory response 20 0.003

GO:0002684~positive regulation of immune system process 30 0.005

GO:0048584~positive regulation of response to stimulus 27 0.001

GO:0050778~positive regulation of immune response 21 0.004

GO:0002253~activation of immune response 15 0.004

GO:0006959~humoral immune response 11 0.007

GO:0045087~innate immune response 16 0.002

GO:0006956~complement activation 9 0.003

GO:0002541~activation of plasma proteins involved in acute inflammatory response 9 0.003

GO:0002252~immune effector proces 17 0.003

GO:0002250~adaptive immune response 12 0.009

GO:0016064~immunoglobulin mediated immune response 10 0.003

GO:0019724~B cell mediated immunity 10 0.004

GO:0002449~lymphocyte mediated immunity 11 0.004

GO:0002443~leukocyte mediated immunity 11 0.001

GO:0051605~protein maturation by peptide bond cleavage 10 0.001

GO:0051604~protein maturation 12 0.002

GO:0002455~humoral immune response mediated by circulating immunoglobulin 6 0.002

GO:0006958~complement activation. classical pathway 6 0.002

GO:0016485~protein processing 11 0.004

Cluster 6

GO:0008009~chemokine activity 12 0.001

GO:0042379~chemokine receptor binding 12 0.001

GO:0005125~cytokine activity 17 0.006

Cluster 7

GO:0048002~antigen processing and presentation of peptide antigen 12 0.009

GO:0002504~antigen processing and presentation of peptide or polysaccharide antigen viaMHC class II 8 0.003

GO:0002478~antigen processing and presentation of exogenous peptide antigen 5 0.004

GO:0019884~antigen processing and presentation of exogenous antigen 5 0.004

GO:0019886~antigen processing and presentation of exogenous peptide antigen via MHCclass II 4 0.001

GO:0002495~antigen processing and presentation of peptide antigen via MHC class II 4 0.001

Cluster 8

GO:0006935~chemotaxis 14 0.004

GO:0007626~locomotory behawior 17 0.001

GO:0050900~leukocyte migration 8 0.001

GO:0030595~leukocyte chemotaxis 6 0.002

GO:0060326~cell chemotaxis 6 0.002

GO:0030593~neutrophil chemotaxis 4 0.002

**Table S3**. Gene cluster differing in expression between the CoNS-1/2 (coagulase-negative*Staphyloccoci* in 1^st^ and 2^nd^ lactation) and H (Healthy) groups in the parenchyma of dairy cattle mammary gland.

Cluster 1 Number of genes p

GO:0006952~defense response 8 0.004

GO:0002526~acute inflammatory response 4 0.006

GO:0006954~inflammatory response 4 0.065

GO:0009611~response to wounding 4 0.203

Cluster 2

GO:0007178~transmembrane receptor protein serine/threonine kinase signaling pathway 4 0.006

GO:0007167~enzyme linked receptor protein signaling pathway 6 0.008

GO:0007179~transforming growth factor beta receptor signaling pathway 3 0.015

GO:0007166~cell surface receptor linked signal transduction 9 0.096

Cluster 3

GO:0030036~actin cytoskeleton organization 4 0.038

GO:0030029~actin filament-based process 4 0.041

GO:0007010~cytoskeleton organization 4 0.018

Cluster 4

GO:0006955~immune response 8 0.015

GO:0042612~MHC class I protein complex 3 0.067

GO:0042611~MHC protein complex 3 0.012

GO:0019882~antigen processing and presentation 3 0.016

GO:0044459~plasma membrane part 7 0.068

**Table S4**. Gene cluster differing in expression between the CoNS-3/4 (coagulase-positive *Staphyloccoci* in 3^rd^ and 4^th^ lactation) and H (Healthy) groups in the parenchyma of dairy cattle mammary gland.

Cluster 1 Number of genes p

GO:0006952~defense response 46 0.000

GO:0006954~inflammatory response 28 0.007

GO:0002526~acute inflammatory response 18 0.004

GO:0009611~response to wounding 31 0.003

GO:0002541~activation of plasma proteins involved in acute inflammatory response 10 0.002

GO:0006956~complement activation 10 0.002

GO:0006959~humoral immune response 11 0.004

GO:0051605~protein maturation by peptide bond cleavage 12 0.002

GO:0051604~protein maturation 14 0.003

GO:0002684~positive regulation of immune system process 21 0.005

GO:0002252~immune effector process 15 0.009

GO:0016485~protein processing 13 0.001

GO:0050778~positive regulation of immune response 15 0.003

GO:0002449~lymphocyte mediated immunity 11 0.004

GO:0002250~adaptive immune response 11 0.004

GO:0016064~immunoglobulin mediated immune response 10 0.004

GO:0048584~positive regulation of response to stimulus 18 0.004

GO:0019724~B cell mediated immunity 10 0.005

GO:0002455~humoral immune response mediated by circulating immunoglobulin 7 0.001

GO:0006958~complement activation. classical pathway 7 0.001

GO:0002443~leukocyte mediated immunity 11 0.001

GO:0002253~activation of immune response 11 0.002

GO:0045087~innate immune response 12 0.004

Cluster 2

GO:0048002~antigen processing and presentation of peptide antigen 11 0.010

GO:0019882~antigen processing and presentation 18 0.003

GO:0002504~antigen processing and presentation of peptide or polysaccharide antigen via MHCclass II 8 0.003

GO:0042611~MHC protein complex 14 0.001

GO:0002474~antigen processing and presentation of peptide antigen via MHC class I 7 0.003

GO:0042613~MHC class II protein complex 7 0.004

GO:0042612~MHC class I protein complex 7 0.004

Cluster 3

GO:0008009~chemokine activity 11 0.005

GO:0042379~chemokine receptor binding 11 0.001

GO:0006935~chemotaxis 12 0.001

GO:0005125~cytokine activity 14 0.001

GO:0007626~locomotory behavior 15 0.001

Cluster 4

GO:0048002~antigen processing and presentation of peptide antigen 11 0.003

GO:0002504~antigen processing and presentation of peptide or polysaccharide antigen via MHCclass II 8 0.005

GO:0002478~antigen processing and presentation of exogenous peptide antigen 5 0.009

GO:0019884~antigen processing and presentation of exogenous antigen 5 0.004

GO:0019886~antigen processing and presentation of exogenous peptide antigen via MHC class II 4 0.005

GO:0002495~antigen processing and presentation of peptide antigen via MHC class II 4 0.005

Cluster 5

GO:0006928~cell motion 16 0.001

GO:0051674~localization of cell 14 0.003

GO:0048870~cell motility 14 0.004

GO:0016477~cell migration 13 0.004

GO:0030595~leukocyte chemotaxis 5 0.004

GO:0060326~cell chemotaxis 5 0.004

GO:0050900~leukocyte migration 6 0.005

GO:0030593~neutrophil chemotaxis 3 0.005
