# Supplementary material for: Transcriptome profiling of Staphylococci-infected cow mammary gland parenchyma
Source: BMC Vet Res. 2017 Jun 6;13:161. doi: 10.1186/s12917-017-1088-2 (PMC5477815; doi:10.1186/s12917-017-1088-2)
Supplement: Supplementary file 11 — Microbiological examination. (DOCX 13 kb) [file 12917_2017_1088_MOESM11_ESM.docx]

**Additional file 11**

Microbiological examination

Milk samples were taken from each quarter of the udder two days before slaughter and examined for the presence of bacteria. The milk was streaked on agar with 5% sheep blood (Columbia, bioMérieux, Craponne, France) and Chapman-Mannitol Salt Agar MSA (bioMérieux, Craponne, France) and incubated at 37°C for 18-24 h. Phenotypic evaluation of isolates included colony morphology, cell morphology and biochemical properties. Production of coagulase by Staphylococci was detected using a tube test with rabbit plasma. Additionally, S. aureus strains were identified using SlidexStaph-Kit (bioMérieux, Craponne, France).
