# Supplementary material for: Transcriptome profiling of Staphylococci-infected cow mammary gland parenchyma
Source: BMC Vet Res. 2017 Jun 6;13:161. doi: 10.1186/s12917-017-1088-2 (PMC5477815; doi:10.1186/s12917-017-1088-2)
Supplement: Supplementary file 12 — Pooling, labeling, and hybridization of RNA sample schemes used in microarray analysis. (DOCX 680 kb) [file 12917_2017_1088_MOESM12_ESM.docx]

**Additional file 12.**

Pooling, labelling and hybridisation of RNA samples schemes in microarray analysis. Red colour – Cyanine3 (Cy3); blue colour – Cyanine5 (Cy5). A) The scheme of analysis for CoPS-1/2 group (coagulase-negative bacteria in milk of cows being in 1^st^ or 2^nd^ lactation); B) The scheme of analysis for CoPS-3/4 group (coagulase-negative bacteria in milk of cows being in 3^rd^or 4^th^lactation); C) The scheme of analysis for CoNS-1/2 group (coagulase-negative bacteria in milk of cows being in 1^st^or 2^nd^lactation); D) The scheme of analysis for CoNS-3/4 group (coagulase-negative bacteria in milk of cows being in 3rd or 4^th^ lactation).


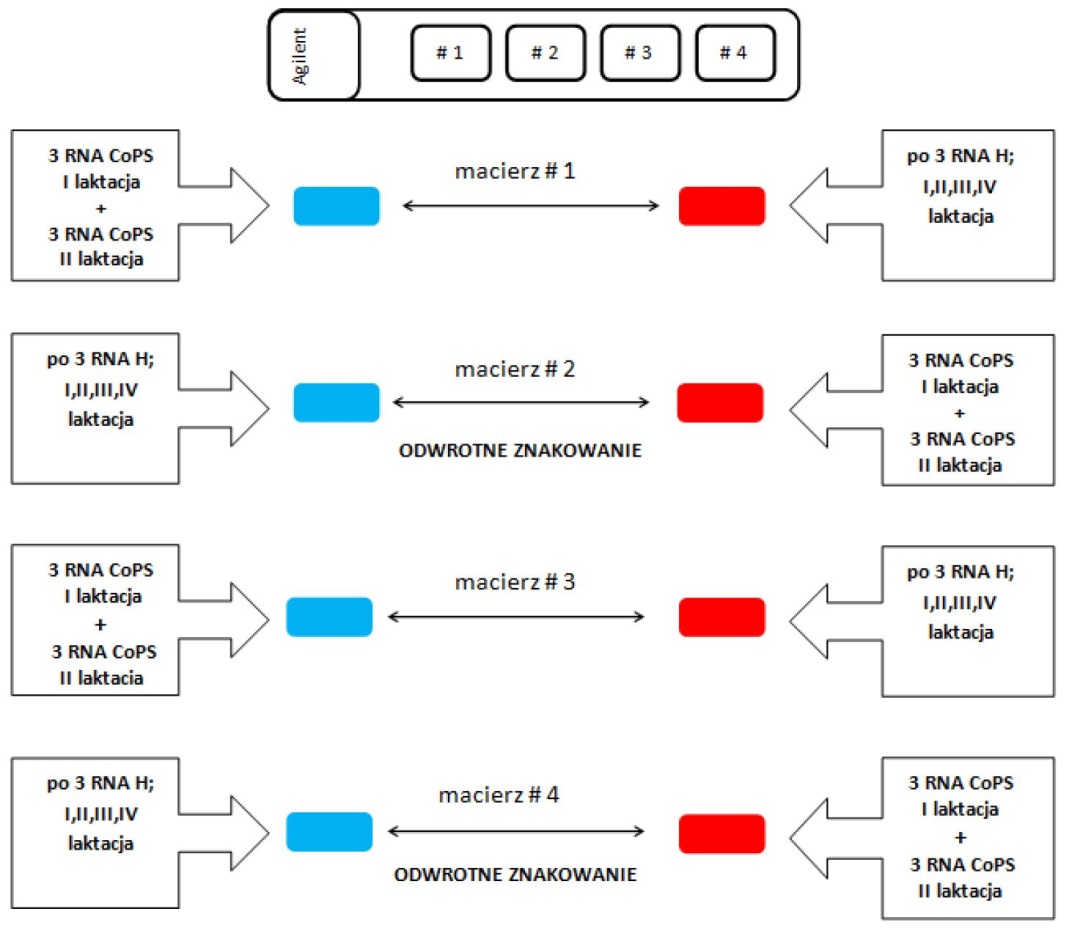


**3 RNA CoPS**

**2^nd^lactation**

**Cy5**

**Cy5**

**Cy5**

**Cy5**

**Cy3**

**Cy3**

**Cy3**

**Cy3**

**3 RNA CoPS**

**1^st^ lactation**

**3 RNA from each H group:**

**From 1^st^ to 4^th^ lactations (N=12)**

**3 RNA from each H group:**

**From 1^st^ to 4^th^ lactations (N=12)**

**3 RNA from each H group:**

**From 1^st^ to 4^th^ lactations (N=12)**

**3 RNA from each H group:**

**From 1^st^ to 4^th^ lactations (N=12)**

**3 RNA CoPS**

**2^nd^ lactation**

**3 RNA CoPS**

**1^st^lactation**

**3 RNA CoPS**

**2^nd^ lactation**

**3 RNA CoPS**

**2^nd^ lactation**

**3 RNA CoPS**

**1^st^ lactation**

**Reverse labelling**

**Reverse labelling**

**microarray**

**microarray**

**microarray**

**microarray**

**3 RNA CoPS**

**1^st^ lactation**

A) The scheme of analysis for CoPS-1/2 group (coagulase-negative bacteria in milk of cows being in 1st or 2nd lactation).


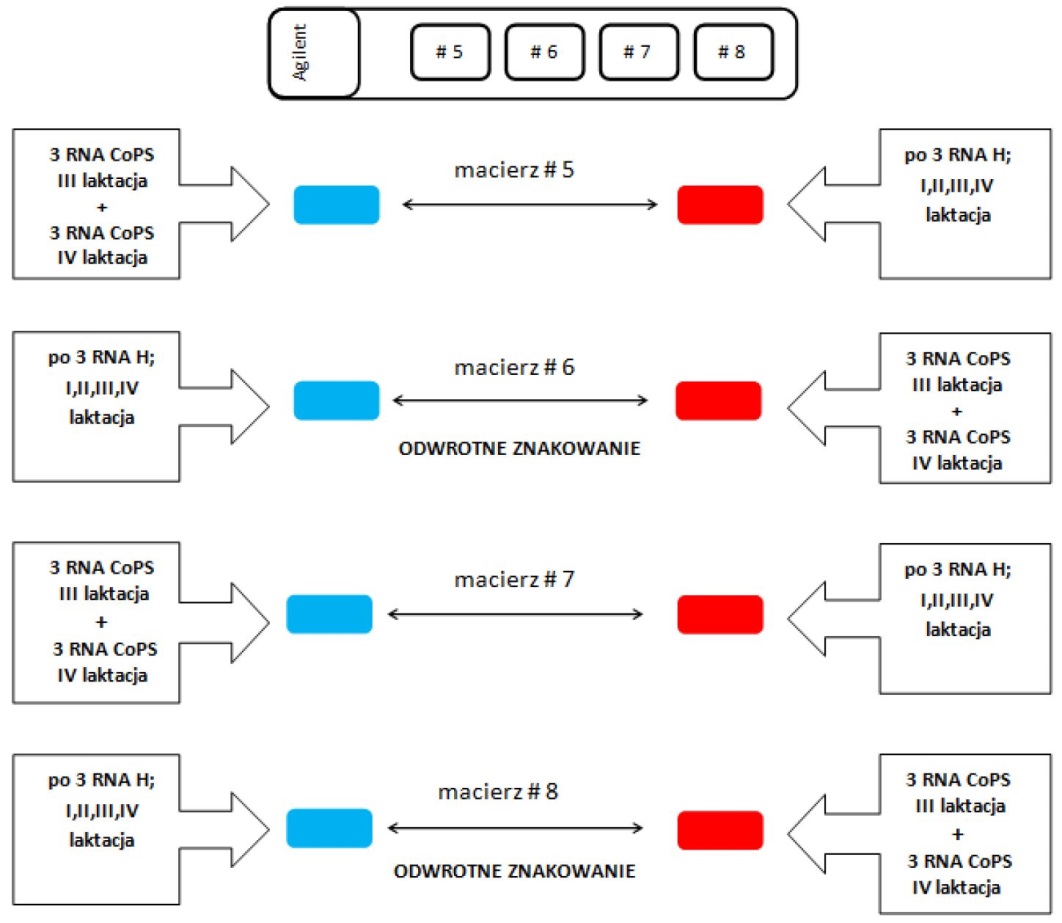


**Cy3**

**Cy3**

**Cy3**

**Cy3**

**Cy5**

**Cy5**

**Cy5**

**Cy5**

**3 RNA CoPS**

**3^rd^ lactation**

**+**

**3 RNA CoPS**

**4^th^ lactation**

**microarray**

**3 RNA CoPS**

**3^rd^ lactation**

**+**

**3 RNA CoPS**

**4^th^ lactation**

**3 RNA CoPS**

**3^rd^ lactation**

**+**

**3 RNA CoPS**

**4^th^ lactation**

**3 RNA CoPS**

**3^rd^ lactation**

**+**

**3 RNA CoPS**

**4^th^ lactation**

**microarray**

**3 RNA from each H group:**

**From 1^st^ to 4^th^ lactations (N=12)**

**3 RNA from each H group:**

**From 1^st^ to 4^th^ lactations (N=12)**

**microarray**

**microarray**

**3 RNA from each H group:**

**From 1^st^ to 4^th^ lactations (N=12)**

**3 RNA from each H group:**

**From 1^st^ to 4^th^ lactations (N=12)**

**Reverse labelling**

**Reverse labelling**

B) The scheme of analysis for CoPS-3/4 group (coagulase-negative bacteria in milk of cows being in 3rd or 4th lactation).


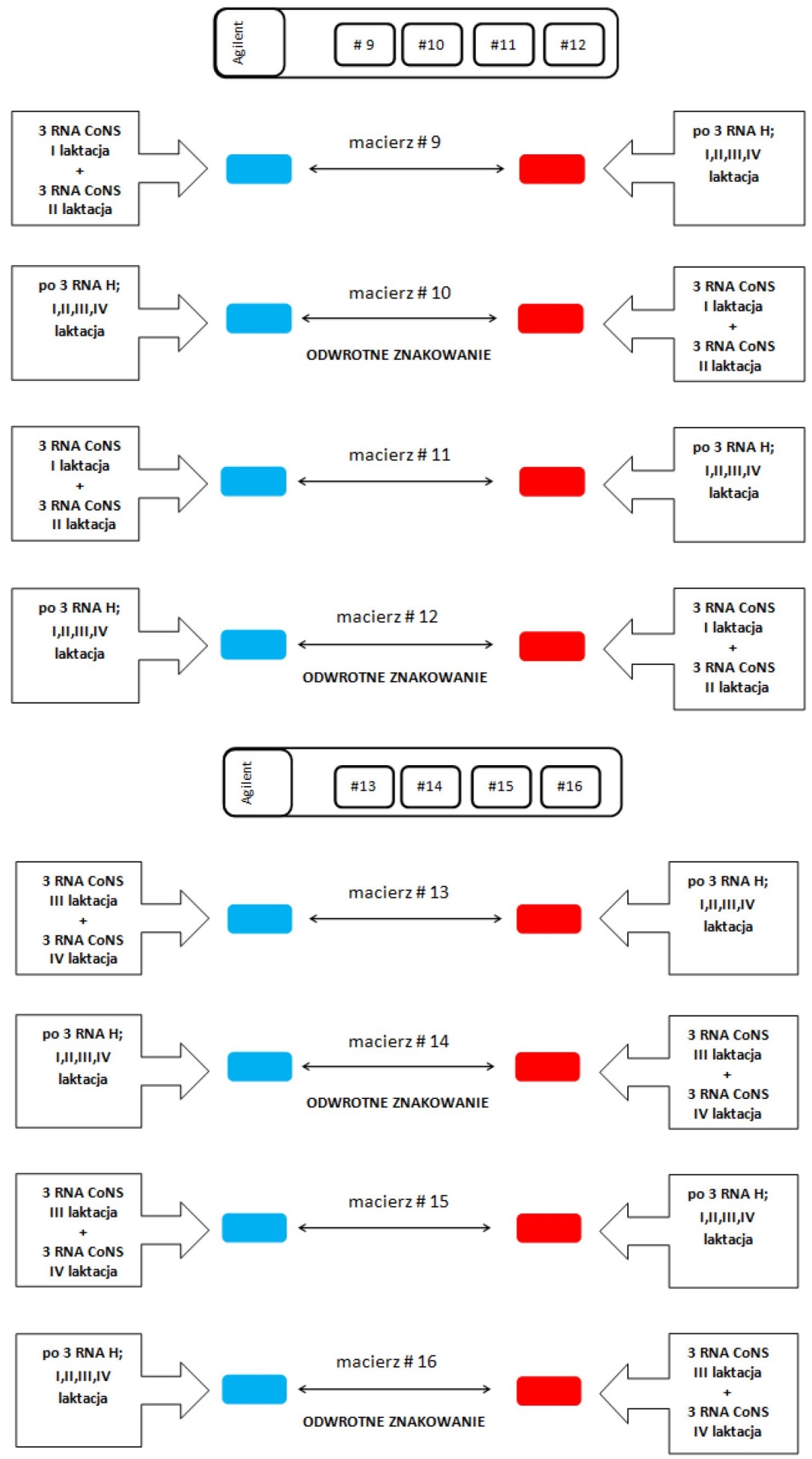


D) The scheme of analysis for CoNS-3/4 group (coagulase-negative bacteria in milk of cows being in 3^rd^or 4^th^lactation).

C) The scheme of analysis for CoNS-1/2 group (coagulase-negative bacteria in milk of cows being in 1^st^or 2^nd^ lactation).

**Cy3**

**Cy3**

**Cy3**

**Cy3**

**Cy3**

**Cy3**

**Cy3**

**Cy3**

**Cy5**

**Cy5**

**Cy5**

**Cy5**

**Cy5**

**Cy5**

**Cy5**

**Cy5**

**microarray**

**microarray**

**Reverse labelling**

**Reverse labelling**

**Reverse labelling**

**Reverse labelling**

**microarray**

**microarray**

**microarray**

**microarray**

**microarray**

**microarray**

**3 RNA CoNS**

**3^rd^ lactation**

**+**

**3 RNA CoNS**

**4^th^ lactation**

**3 RNA CoNS**

**3^rd^ lactation**

**+**

**3 RNA CoNS**

**4^th^ lactation**

**3 RNA CoNS**

**3^rd^ lactation**

**+**

**3 RNA CoNS**

**4^th^ lactation**

**3 RNA CoNS**

**3^rd^ lactation**

**+**

**3 RNA CoNS**

**4^th^ lactation**

**3 RNA CoNS**

**1^st^ lactation**

**+**

**3 RNA CoNS**

**2^nd^ lactation**

**3 RNA CoNS**

**^1st^ lactation**

**+**

**3 RNA CoNS**

**2^nd^ lactation**

**3 RNA CoNS**

**^1st^ lactation**

**+**

**3 RNA CoNS**

**2^nd^ lactation**

**3 RNA CoNS**

**^1st^ lactation**

**+**

**3 RNA CoNS**

**2^nd^ lactation**

**3 RNA from each H group:**

**From 1^st^ to 4^th^ lactations (N=12)**

**3 RNA from each H group:**

**From 1^st^ to 4^th^ lactations (N=12)**

**3 RNA from each H group:**

**From 1^st^ to 4^th^ lactations (N=12)**

**3 RNA from each H group:**

**From 1^st^ to 4^th^ lactations (N=12)**

**3 RNA from each H group:**

**From 1^st^ to 4^th^ lactations (N=12)**

**3 RNA from each H group:**

**From 1^st^ to 4^th^ lactations (N=12)**

**3 RNA from each H group:**

**From 1^st^ to 4^th^ lactations (N=12)**

**3 RNA from each H group:**

**From 1^st^ to 4^th^ lactations (N=12)**
