# Supplementary material for: Transcriptome profiling of Staphylococci-infected cow mammary gland parenchyma
Source: BMC Vet Res. 2017 Jun 6;13:161. doi: 10.1186/s12917-017-1088-2 (PMC5477815; doi:10.1186/s12917-017-1088-2)
Supplement: Supplementary file 13 — The primer sequences, amplicon length, melting temperature and no. of GenBank access of housekeeping genes examined to use in qPCR analysis. Table S6. The primer sequences, amplicon length, melting temperature and No. of GenBank access of validated genes. (DOCX 21 kb) [file 12917_2017_1088_MOESM13_ESM.docx]

**Additional file 12**

GeNorm software [1] was used to identify the most stable reference genes using the Crossing Point (CP) values and considering the qPCR efficiency (E). Amplification efficiency was calculated based on the slope of the standard curve using the formula E=10^(-1/slope)^. Ct and E values were entered into the geNorm applet, which then ranked the genes basing on M-values, where the gene with the most stable expression has the lowest M-value [2]. This measure is based on the principle that the expression ratio of 2 ideal control genes is identical in all samples, regardless of the experimental conditions. For the two HK genes with the lowest M-values the Normalization Factor (NF) was calculated to establish the relative expression of target genes. Relative mRNA expression of target genes was calculated based on HKG NFs and using the mathematical model for relative quantification in qPCR described by Pfaffl [3] (2001).

**Table S5.** The primer sequences, amplicon length, melting temperature and no. of GenBank access of housekeeping genes examined to use in qPCR analysis

| Gene name | Gene Symbol | Biological function | Primer sequence | Accession number from GenBank | Ampliconlength(bp) | Melting temp. (°C) |
| --- | --- | --- | --- | --- | --- | --- |
| Β-actin | *ACTB* | Cytoskeletalstructural protein | GAGCGGGAAATCGTCCGTGAC  GTGTTGGCGTAGAGGTCCTTGC | NC_007326 | 278 | 60 |
| Glyceraldehyde-3P-dehydrogenase | *GAPDH* | Oxidoreductase in glucose metabolism | ACCACTTTGGCATCGTGGAG  GGGCCATCCACAGTCTTCTG | U85042 | 75 | 58 |
| Succinate dehydrogenase complex subunit A | *SDHA* | Catalyzes the oxidation of succinate | GCAGAACCTGATGCTTTGTG  CGTAGGAGAGCGTGTGCTT | NC_007318 | 185 | 60 |
| TATA box-binding protein | *TATABP* | Transcription factor | ACAACAGCCTCCCACCCTATGC  GTGGAGTCAGTCCTGTGCCGTAA | NM_001075742 | 111 | 60 |
| Zeta polypeptide | *YWHAZ* | Signal transduction by binding to phosphoserine-containing protein | GCATCCCACAGACTATTTCC  GCAAAGACAATGACAGACCA | NW_001493253 | 120 | 60 |
| Hypoxanthine phosphoribosyltransferase1 | *HPRT1* | Enzyme which plays a central role in the generation of purine nucleotides through the purine salvage pathway | TGCTGAGGATTTGGAGAAGG CAACAGGTCGGCAAAGAACT | NW_001501830 | 154 | 58 |

**Table S6.** The primer sequences, amplicon length, melting temperature and No. of GenBank access of validated genes

| Gene name | Gene symbol | Primers 5'‐3' | Amplikon lenght (bp) | Temperature annealing  (°C) | Access No. GenBank |
| --- | --- | --- | --- | --- | --- |
| Carbonic anhydrase 4 | CA4 | GTGCACGAGAAAGAGAAGGG  GCGGAAGTAGTGCCTCAGAC | 234 | 60 | NM_173897.1 |
| Carbonic anhydrase 6 | CA6 | TGCAGGACAAAGCACAGTTC  TGCTACCACAAACCAGTGGA | 136 | 60 | NM_173898.2 |
| complement factor B | CFB* | CTTCATTCAAGTTGGCGTGA  GCAGCACCTGGTAGAGGTTG | 110 | 58 | NM_001040526.1 |
| Ceruloplasmin (ferroxidase) | CP* | TTCATGCACATGGAATGACTT  TAAAGGCCCAATGAGTCCTG | 236 | 58 | XM_592003 |
| Glycerol-3-phosphate acyltransferase | GPAM | TACGGATGTGTCAAAACGGA  GCTTGCTCCAAAGAAAGTGG | 104 | 60 | NM_001012282.1 |
| Haptoglobin | HP* | TGGTCTCCCAGCATAACCT  AGGGTGGAGAACCACCTTCT | 185 | 58 | NM_001040470 |
| Interleukin 8 | IL8* | AGAACTTCGATGCCAATGCAT  GGGTTTAGGCAGACCTCGTTT | 150 | 60 | NM_173925 |
| Ryanodine receptor type 3 | RYR3 | CAAAGCACAAGATGCCAAGA  CTGAATAAGGCGGTCGTGTT | 159 | 60 | XM_003584950.1 |
| Serum amyloid A3 | SAA3* | CTCAAGGAAGCTGGTCAAGG  CTTCGAATCCTCCCGTACCT | 240 | 58 | NM_181016 |
| UL16 binding protein 3 | ULBP3 | CAGCAGTTACAGCACCTGGA  GAGTCCTGAGGCCAACAGAG | 143 | 60 | NM_001103233.1 |

* sequence of peimers according to Whelehan et al. (2011)

** sequence of peimers according to Moyes et al. (2009)

1. Lisowski P, Pierzchała M, Gościk J, PareekChS, Zwierzchowski L: Evaluation of reference genes for studies of gene expression in the bovine liver, kidney, pituitary, and thyroid. J Appl Gene 2008, 49:367–372.

2. Vandesompele J, De Preter K, Pattyn F, Poppe B, Van Roy N, De Paepe A, Speleman F: Accurate normalization of real-time quantitative RT-PCR data by geometric averaging of multiple internal control genes. Genome Biology 2002, 3, RESEARCH0034.

3. Pfaffl MW: A new mathematical model for relative quantification in real time RT-PCR. Nucleic Acids Res 2001, 29: e45.

4. Moyes KM, Drackley JK, Morin DE, Bionaz M, Rodriguez-Zas SL, Everts RE, Lewin HA, Loor JJ. 2009. Gene network and pathway analysis of bovine mammary tissue challenged with Streptococcus uberis reveals induction of cell proliferation and inhibition of PPAR gamma signaling as potential mechanism for the negative relationships between immune response and lipid metabolism. BMC Genomics 10, 542

5. Whelehan CJ, Meade KG, Eckersall PD, Young FJ, O'Farrelly C. 2011. Experimental Staphylococcus aureus infection of the mammary gland induces region-specific changes in innate immune gene expression. Vet Immunol Immunopathol 2011 15;140(3-4):181-189.
