# Supplementary material for: Transcriptome profiling of Staphylococci-infected cow mammary gland parenchyma
Source: BMC Vet Res. 2017 Jun 6;13:161. doi: 10.1186/s12917-017-1088-2 (PMC5477815; doi:10.1186/s12917-017-1088-2)
Supplement: Supplementary file 14 — Data normalization and selection of differently expressed genes (DEGs) (DOCX 15 kb) [file 12917_2017_1088_MOESM14_ESM.docx]

**Additional file 14**

Data normalization and selection of differently expressed genes (DEGs)

Raw data were quantile normalized and the average signal intensities were analyzed in Partek Genomic Suite (Partek, Inc.) v. 6.6 after Log2 transformation. Qualitative analysis was performed to identify outliers and artifacts on the microarray. Two-way ANOVA (Analysis of Variance) was performed. Lists of significantly and differentially expressed genes between experimental groups were generated with the cutoff values: p-value with FDR < 0.05, -1.5>Fold Change>1.5). Fisher's Least Significant Difference (LSD) was used as the contrast method to compare control vs infected groups. To identify biological processes, molecular functions and biochemical pathways in which the differentially expressed genes could be involved, the Database for Annotation, Visualization and Integrated Discovery (DAVID) was used (http://david.abcc.ncifcrf.gov/home.jsp/). Enrichment of the gene lists was made using GeneOntology (GO) (http://www.genontology.org/) and the Kyoto Encyclopedia of Genes and Genomes (KEGG) (http://www.genome.jp/kegg/pathway.html/), and using Ingenuity®Pathway Analysis (IPA) (Ingenuity® Systems, USA).

For functional clusterings the identified GO categories have been subjected to the DAVID Functional Annotation Clustering module. To design functional networks of gene expression, the entire lists of genes, along with FoldChange and P values, were introduced into Ingenuity Pathway Analysis (IPA) software. As IDs of genes, GenBank accession numbers of the human homologs of genes present in the list of the Bovine (V2) microarray were used. The set of these genes was used as the reference group for statistical IPA analysis (Ingenuity®Pathway Analysis). Microarray data was deposited in the GEO database and is available under the GEO accession number GSE34031.
